# A Global Survey of Hypervirulent Aeromonas hydrophila (vAh) Identified vAh Strains in the Lower Mekong River Basin and Diverse Opportunistic Pathogens from Farmed Fish and Other Environmental Sources

**DOI:** 10.1128/spectrum.03705-22

**Published:** 2023-02-23

**Authors:** Tingbi Xu, Cody R. Rasmussen-Ivey, Francesco S. Moen, Ana Fernández-Bravo, Brigitte Lamy, Roxana Beaz-Hidalgo, Chan Dara Khan, Graciela Castro Escarpulli, Ina Salwany M. Yasin, Maria J. Figueras, Mohamad Azzam-Sayuti, Muhammad Manjurul Karim, K. M. Mazharul Alam, Thao Thu Thi Le, Ngo Huynh Phuong Thao, Samuel Addo, Samuel Duodu, Shahzad Ali, Tooba Latif, Sothea Mey, Thay Somony, Mark R. Liles

**Affiliations:** a Department of Biological Sciences, Auburn University, Alabama, USA; b Department of Biology, Tufts University, Medford, Massachusetts, USA; c Unit of Microbiology, Department of Basic Health Sciences, Faculty of Medicine and Health Sciences, IISPV, University Rovira i Virgili, Reus, Spain; d INSERM U1065, Laboratoire de Bactériologie, CHU Nice, Faculté de Médecine, Université Côte d’Azur, Nice, France; e Centre for Molecular Bacteriology and Infection, Imperial College of London, London, United Kingdom; f Aquatic Animal Health and Disease Management Office, Department of Aquaculture Development, Fisheries Administration, Ministry of Agriculture Forestry and Fisheries, Phnom Penh, Cambodia; g Laboratorio de Investigación Clínica y Ambiental, Escuela Nacional de Ciencias Biológicas, Instituto Politécnico Nacional, Ciudad de México, Mexico; h Department of Aquaculture, Universiti Putra Malaysia, Serdang, Selangor, Malaysia; i Institute of Bioscience, Universiti Putra Malaysia, Serdang, Selangor, Malaysia; j Department of Microbiology, University of Dhaka, Dhaka, Bangladesh; k Division of Aquacultural Biotechnology, Biotechnology Center of Ho Chi Minh City, Ho Chi Minh City, Vietnam; l Department of Marine and Fisheries Sciences, University of Ghana, Legon, Ghana; m Department of Biochemistry, Cell, and Molecular Biology, University of Ghana, Legon, Ghana; n Wildlife Epidemiology and Molecular Microbiology Laboratory, Department of Wildlife and Ecology, University of Veterinary and Animal Sciences, Lahore, Pattoki, Pakistan; USGS, Eastern Ecological Science Center

**Keywords:** *Aeromonas hydrophila*, pathogen, freshwater fish, pandemic, comparative genomics, worldwide

## Abstract

Hypervirulent Aeromonas hydrophila (vAh) has emerged as the etiologic agent of epidemic outbreaks of motile *Aeromonas* septicemia (MAS) in high-density aquaculture of farmed carp in China and catfish in the United States, which has caused millions of tons of lost fish. We conducted a global survey to better understand the evolution, geographical distribution, and phylogeny of vAh. *Aeromonas* isolates were isolated from fish that showed clinical symptoms of MAS, and pure cultures were screened for the ability to utilize *myo*-inositol as the sole carbon source. A total of 113 *myo-*inositol-utilizing bacterial strains were included in this study, including additional strains obtained from previously published culture collections. Based on a *gyrB* phylogeny, this collection included 66 A. hydrophila isolates, 48 of which were vAh. This collection also included five new vAh isolates from diseased Pangas catfish (Pangasius pangasius) and striped catfish (Pangasianodon hypophthalmus) obtained in Cambodia and Vietnam, respectively. Genome sequences were generated from representative vAh and non-vAh isolates to evaluate the potential for lateral genetic transfer of the *myo-*inositol catabolism pathway. Phylogenetic analyses of each of the nine genes required for *myo*-inositol utilization revealed the close affiliation of vAh strains regardless of geographic origin and suggested lateral genetic transfer of this catabolic pathway from an Enterobacter species. Prediction of virulence factors was conducted to determine differences between vAh and non-vAh strains in terms of virulence and secretion systems. Core genome phylogenetic analyses on vAh isolates and *Aeromonas* spp. disease isolates (55 in total) were conducted to evaluate the evolutionary relationships among vAh and other *Aeromonas* sp. isolates, which supported the clonal nature of vAh isolates.

**IMPORTANCE** This global survey of vAh brought together scientists that study fish disease to evaluate the evolution, geographical distribution, phylogeny, and hosts of vAh and other *Aeromonas* sp. isolates. In addition to vAh isolates from China and the United States, four new vAh isolates were isolated from the lower Mekong River basin in Cambodia and Vietnam, indicating the significant threat of vAh to modern aquaculture and the need for improved biosecurity to prevent vAh spread.

## INTRODUCTION

*Aeromonas* species are ubiquitous in aquatic habitats and can be found in both fresh and brackish water (1). Aeromonas hydrophila is one of the most well-known pathogenic species within the genus *Aeromonas* and is known for its high tolerance to extremes of temperature, pH, and salinity that enable it to flourish in a variety of environments and to be an opportunistic pathogen in a diverse range of hosts, including fish, amphibians, birds, reptiles, and mammals (2–6). Typically, A. hydrophila causes motile *Aeromonas* septicemia (MAS) in fish that are infected with other primary pathogens, such as Flavobacterium columnare, or are under stress due to harsh environmental conditions and/or high-density farming (7, 8). MAS is associated with high mortality within a short time, and infected fish generally show a variety of symptoms, such as hemorrhaging and lesions on the fish surface (9). A. hydrophila disease isolates are known to have significant antigenic diversity, with more than 40 O-antigen serotypes observed (10, 11).

The epidemic outbreaks among farmed fish due to hypervirulent A. hydrophila (vAh) have been notable for their rapid emergence and high mortality (12, 13). The first isolated strain of this deadly A. hydrophila pathotype, J-1, was obtained in Jiangsu province, China, from an epidemic outbreak of MAS that resulted in high mortality in cultured carp and bream in 1989 (14). Outbreaks of MAS caused by vAh were reported again in Jiangsu and in Guangdong and Fujian provinces in 2010 (15), resulting in about 2,200 tons of fish losses per year in China (16, 17). The isolated vAh strains J-1, NJ-35, and ZC1 were all categorized as sequence type 251 (ST251), and were found to be clonal based on genome sequence analyses (12).

The first report of a vAh isolate in the United States was strain S04-690, which was isolated from channel catfish (Ictalurus punctatus) in Mississippi in 2004 (4). The first major MAS outbreak in the United States was in catfish production ponds in western Alabama in 2009, from which vAh strain ML09-119 was isolated. The MAS outbreaks due to vAh in Alabama have continued, causing the loss of more than 5,000 tons of farmed channel catfish each year, and the current accumulative loss of farmed channel catfish due to vAh in the state of Alabama is estimated to be over 40 million pounds (Anita Kelly, unpublished data) (18). Due to the lack of effective control, vAh is a consistent threat to U.S. and Chinese aquaculture, and many more countries could be affected. To date, there have not been any vAh global surveillance efforts.

Previous studies of vAh isolated from carp in China or from catfish in the United States indicate that these strains share a recent common ancestor and have common features, such as the ability to utilize *myo*-inositol as the sole carbon source (19). Based on phylogenetic analysis of vAh-specific gene sequences, vAh isolates from carp are at the root of the vAh tree, suggesting that the emergence of vAh in the United States was due to importation of live carp species or fish products from Asia (4). Beginning in the 1960s, silver carp (Hypophthalmichthys molitrix) and bighead carp (Hypophthalmichthys nobilis) were introduced into U.S. catfish ponds to control algal blooms. Massive flooding of the Mississippi River in 1993 resulted in the release of Asian carp into the Mississippi River basin, where these invasive carp species have continued to spread and are a major threat to the Great Lakes ecosystem (20). The importation of bighead carp into the United States is now prohibited based on the Asian Carp Prevention and Control Act signed into law in 2010. The global value of trade in exporting live carp was estimated at $164 million in 2020, with China being the world leader in live carp export at $103 million per year (21). The lack of sufficient biosecurity measures to prevent the spread of vAh-infected fish prompted the need for a global survey to assess vAh dissemination among various farmed fish species in different regions of the world.

Despite the evidence that vAh strains are clonal and have recently spread from Asia to the United States, there are some genetic differences among vAh strains. In particular, while vAh isolates from carp species in China typically have a complete type VI secretion system (T6SS) (12, 22), most vAh isolates from channel catfish in the United States, and especially from western Alabama, lack a complete T6SS and only carry *hcp1*, *tssH*, and *vgrG1* (23). The carp vAh isolate NJ-35, which has a complete T6SS, has been found to express a phospholipase that contributes to biofilm formation and virulence in zebrafish (Danio rerio) (24). While lacking many T6SS-associated genes, the presence of *hcp1* and *vgrG1* have been found to contribute to vAh ML09-119 virulence (23), but the degree to which the T6SS plays a role in fish host specificity and virulence has yet to be defined. The evolution of vAh strains as they infect and replicate in different fish species is of significant interest. Our lack of knowledge regarding fish host range and geographic distribution also prompted us to conduct a vAh global survey. As a group of fish disease experts from around the world, we primarily sampled freshwater fish with disease symptoms characteristic of MAS and obtained pure bacterial cultures that were evaluated for growth on *myo*-inositol, a phenotype that has been consistent in vAh strains isolated from China and the United States. A phylogenetic analysis of *myo*-inositol-utilizing strains using *gyrB* sequences was conducted to further characterize disease isolates. Finally, for representative vAh and non-vAh strains, we conducted comparative genome analyses to provide further information on the phylogeny and predicted virulence factors of vAh strains. This study is a first step toward a better understanding of vAh worldwide distribution, uniting fish disease researchers in a network that can help track the distribution of vAh and developing methods to protect farmed fish against this emerging pathogen.

## RESULTS AND DISCUSSION

### Identification of *myo*-inositol-utilizing *Aeromonas* sp. strains.

This global vAh survey relied upon an extensive network of microbiologists willing to participate in screening fish disease isolates and cryopreserved collections for the presence of *myo-*inositol utilizing A. hydrophila strains. There have been no previous reports of A. hydrophila strains with the ability to use *myo-*inositol as the sole carbon source other than vAh strains (i.e., ST251). Therefore, by screening bacterial isolates for growth on *myo*-inositol in a minimal medium, our goal was to rapidly and cost-effectively identify putative vAh strains from diverse locales. From this extensive survey, 43 *myo-*inositol-utilizing *Aeromonas* sp. strains were isolated from Pabda (Ompok pabda) from Bangladesh, Pangas catfish from Cambodia, lake water from Finland, Koi (Cyprinus rubrofuscus) from France, basa fish (Pangasius bocourti) from Malaysia, rainbow trout (Oncorhynchus mykiss) from Mexico, crab (*Brachyura* spp.) from Norway, trout (*Oncorhynchus* spp.) and human feces from Spain, and striped catfish from Vietnam (Table 1). Typical vAh strains cultured on tryptic soy agar (TSA) produce smooth, rounded, opaque colonies that have a light yellow color with a 2- to 3-mm diameter range after 24 h of incubation (25). The strains that showed a colony morphology consistent with vAh and evident growth on *myo*-inositol (i.e., increase in the optical density at 600 nm [OD_600_] of >0.4 over 48 h) were further validated by molecular phylogenetic analyses (26, 27).

**TABLE 1:** Bacterial isolates used in this study

Strain ID	Country of isolation	Pathotype	Isolation source	GenBank species assignation	Species based on phylogeny and ANI	Accession ID	Reference or source
AL09-71	USA	vAh	Channel catfish	A. hydrophila	A. hydrophila	NZ_CP007566.1	58
AL09-79	USA	vAh	Channel catfish	A. hydrophila	A. hydrophila	NZ_LRRV00000000.1	47
ALG15-098	USA	vAh	Channel catfish	A. hydrophila	A. hydrophila	SAMN05223361	12
IPRS15-28	USA	vAh	Channel catfish	A. hydrophila	A. hydrophila	SAMN05223362	12
J-1	China (P.R.C.)	vAh	Crucian carp	A. hydrophila	A. hydrophila	NZ_CP006883.1	16
JBN2301	China (P.R.C.)	vAh	Crucian carp	A. hydrophila	A. hydrophila	NZ_CP013178.1	59
ML09-119	USA	vAh	Channel catfish	A. hydrophila	A. hydrophila	NC_021290.1	60
ML09-121	USA	vAh	Channel catfish	A. hydrophila	A. hydrophila	NZ_LRRX00000000.1	47
ML09-122	USA	vAh	Channel catfish	A. hydrophila	A. hydrophila	NZ_LRRY00000000.1	47
ML10-51K	USA	vAh	Channel catfish	A. hydrophila	A. hydrophila	SAMN05223363	12
NJ-35	China (P.R.C.)	vAh	Crucian carp	A. hydrophila	A. hydrophila	NZ_CP006870.1	16
GYK1	China (P.R.C.)	vAh	Mandarin fish	A. hydrophila	A. hydrophila	NZ_CP016392.1	61
D4	China (P.R.C.)	vAh	Blunt-snout bream	A. hydrophila	A. hydrophila	NZ_CP013965.1	62
PB10-118	USA	vAh	Channel catfish	A. hydrophila	A. hydrophila	SAMN01085622	47
pc104A	USA	vAh	Channel catfish	A. hydrophila	A. hydrophila	NZ_CP007576.1	58
S04-690	USA	vAh	Channel catfish	A. hydrophila	A. hydrophila	SAMN02404466	4
S13-612	USA	vAh	Channel catfish	A. hydrophila	A. hydrophila	SAMN05292362	12
S13-700	USA	vAh	Channel catfish	A. hydrophila	A. hydrophila	SAMN05292363	12
S14-296	USA	vAh	Channel catfish	A. hydrophila	A. hydrophila	SAMN05292365	12
S14-452	USA	vAh	Channel catfish	A. hydrophila	A. hydrophila	SAMN05256776	12
S14-458	USA	vAh	Channel catfish	A. hydrophila	A. hydrophila	SAMN05223364	12
S14-606	USA	vAh	Channel catfish	A. hydrophila	A. hydrophila	SAMN05292366	12
S15-130	USA	vAh	Channel catfish	A. hydrophila	A. hydrophila	SAMN05223365	12
S15-400	USA	vAh	Channel catfish	A. hydrophila	A. hydrophila	SAMN05223367	12
ZC1	China (P.R.C.)	vAh	Grass carp	A. hydrophila	A. hydrophila	SAMN02404465	4
AL10-121	USA	vAh	Channel catfish	A. hydrophila	A. hydrophila	NZ_LRRW00000000.1	63
AL09-80	USA	vAh	Channel catfish	A. hydrophila	A. hydrophila	JX275838	27
G3	China (P.R.C.)	vAh	Mandarin fish	A. hydrophila	A. hydrophila	KX822741.1	64
AH11P	USA	vAh	Catfish	A. hydrophila	A. hydrophila	KC133524.1	65
IB102	China (P.R.C.)	vAh	Carp	A. hydrophila	A. hydrophila	JQ085433.1	66
JG102	China (P.R.C.)	vAh	Carp	A. hydrophila	A. hydrophila	JQ085448.1	66
JG103	China (P.R.C.)	vAh	Carp	A. hydrophila	A. hydrophila	JQ085449.1	66
JG101	China (P.R.C.)	vAh	Carp	A. hydrophila	A. hydrophila	JN177329.1	66
DLNG201	China (P.R.C.)	vAh	Carp	A. hydrophila	A. hydrophila	JQ085458.1	66
XX-52	China (P.R.C.)	vAh	Carp	A. hydrophila	A. hydrophila	JX025794.1	67
XX-22	China (P.R.C.)	vAh	Carp	A. hydrophila	A. hydrophila	JX025792.1	67
4LNG202	China (P.R.C.)	vAh	Carp	A. hydrophila	A. hydrophila	JQ085443.1	66
4LNS301	China (P.R.C.)	vAh	Carp	A. hydrophila	A. hydrophila	JN177325.1	66
4LNG102	China (P.R.C.)	vAh	Carp	A. hydrophila	A. hydrophila	JQ085441.1	66
PW06	China (P.R.C.)	vAh	Carp	A. hydrophila	A. hydrophila	JN177338.1	66
DBHS101	China (P.R.C.)	vAh	Carp	A. hydrophila	A. hydrophila	JQ085454.1	66
2JBN302	China (P.R.C.)	vAh	Carp	A. hydrophila	A. hydrophila	JQ085474.1	66
2JBN103	China (P.R.C.)	vAh	Carp	A. hydrophila	A. hydrophila	JQ085472.1	66
DLNG102	China (P.R.C.)	vAh	Carp	A. hydrophila	A. hydrophila	JQ085457.1	66
2JBN102	China (P.R.C.)	vAh	Carp	A. hydrophila	A. hydrophila	JQ085471.1	66
2JFN201	China (P.R.C.)	vAh	Carp	A. hydrophila	A. hydrophila	JQ085476.1	66
DLNG202	China (P.R.C.)	vAh	Carp	A. hydrophila	A. hydrophila	JQ085459.1	66
LNB103	China (P.R.C.)	vAh	Carp	A. hydrophila	A. hydrophila	JQ085451.1	66
PW14	China (P.R.C.)	vAh	Carp	A. hydrophila	A. hydrophila	JQ085452.1	66
DBHS102	China (P.R.C.)	vAh	Carp	A. hydrophila	A. hydrophila	JQ085455.1	66
XX-58	China (P.R.C.)	vAh	Carp	A. hydrophila	A. hydrophila	JX025795.1	67
AL09-77	USA	vAh	Channel catfish	A. hydrophila	A. hydrophila	JX275844.1	27
XX-14	China (P.R.C.)	vAh	Carp	A. hydrophila	A. hydrophila	JX025791.1	67
AL09-138	USA	vAh	Channel catfish	A. hydrophila	A. hydrophila	JX275841.1	27
AL10-13	USA	vAh	Channel catfish	A. hydrophila	A. hydrophila	JX275833.1	27
ML09-139	USA	vAh	Channel catfish	A. hydrophila	A. hydrophila	JX275834.1	27
AL09-74	USA	vAh	Channel catfish	A. hydrophila	A. hydrophila	KF913679.1	4
CPF2-S1	Cambodia	vAh	Pangas catfish	A. hydrophila	A. hydrophila	JANLOJ000000000	This study
DT-TKT-2020-677	Vietnam	vAh	Striped catfish	A. hydrophila	A. hydrophila	OP198653	This study
DT-TKT-2020-680	Vietnam	vAh	Striped catfish	A. hydrophila	A. hydrophila	OP198652	This study
DT-TKT-2020-681	Vietnam	vAh	Striped catfish	A. hydrophila	A. hydrophila	OP198651	This study
DT-TTD-2020-734	Vietnam	vAh	Striped catfish	A. hydrophila	A. hydrophila	NZ_JALRNI010000001.1	This study
VL-2013-869	Vietnam	non-vAh	Striped catfish	A. hydrophila	A. hydrophila	NZ_JALRNJ000000000.1	This study
BT-2012-871	Vietnam	non-vAh	Striped catfish	A. hydrophila	A. hydrophila	NZ_JALRNL000000000.1	This study
VL-2012-870	Vietnam	non-vAh	Striped catfish	A. hydrophila	A. hydrophila	NZ_JALRNK000000000.1	This study
Ae34	Japan	non-vAh	Koi carp	A. hydrophila	A. hydrophila	NZ_BAXY00000000.1	68
AD9	USA	non-vAh	Alga	A. hydrophila	A. hydrophila	NZ_JFJO00000000.1	69
ATCC 7966	USA	non-vAh	Milk	A. hydrophila	A. hydrophila	CP000462	70
ESV-357	Mexico	non-vAh	Rainbow trout	A. hydrophila	A. hydrophila	KJ743520.1	71
ESV-371	Mexico	non-vAh	Rainbow trout	A. hydrophila	A. hydrophila	KJ743529.1	71
ESV-381	Mexico	non-vAh	Rainbow trout	A. hydrophila	A. hydrophila	KJ743537.1	71
ESV-394	Mexico	non-vAh	Rainbow trout	A. hydrophila	A. hydrophila	KJ743549.1	71
ESV-399	Mexico	non-vAh	Rainbow trout	A. hydrophila	A. hydrophila	KJ743514.1	71
0.14	Spain	non-vAh	Oscar	A. hydrophila	*A. sobria*	JANLFC000000000	This study
14	Malaysia	non-vAh	Clinical	A. hydrophila	*A. dhakensis*	NZ_AOBM00000000.1	72
D69555	Spain	non-vAh	Human feces	A. hydrophila	A. hydrophila	OP198650	This study
2006 4153	Spain	non-vAh	Human hemoculture	A. hydrophila	A. hydrophila	OP198649	This study
AE210	Finland	non-vAh	FW lake water	A. hydrophila	A. hydrophila	JN711784.1	73
AH10	China (P.R.C.)	non-vAh	Grass carp	A. hydrophila	A. hydrophila	NZ_CP011100.1	74
TN97-08	USA	non-vAh	Bluegill	A. hydrophila	*A. hydrophila*	NZ_LNUR00000000.1	47
AHNIH1	USA	non-vAh	Human tissue	A. hydrophila	A. hydrophila	NZ_CP016380.1	75
MN98-04	USA	non-vAh	Tilapia	A. hydrophila	A. hydrophila	SAMN04967900	47
AL97-91	USA	non-vAh	Channel catfish	A. hydrophila	*A. hydrophila*	SAMN04967787	47
AL06-06	USA	non-vAh	Goldfish	*A. hydrophila*	*A. hydrophila*	NZ_CP010947.1	23
AL10-121	USA	non-vAh	Channel catfish	*A. hydrophila*	*A. hydrophila*	NZ_LRRW00000000.1	47
116	Malaysia	non-vAh	Clinical	*A. hydrophila*	*A. dhakensis*	NZ_ANPN00000000.1	72
173	Malaysia	non-vAh	Clinical	*A. hydrophila*	*A. dhakensis*	NZ_AOBN00000000.1	72
187	Malaysia	non-vAh	Clinical	*A. hydrophila*	*A. dhakensis*	NZ_AOBO00000000.1	72
226	Malaysia	non-vAh	Clinical	*A. hydrophila*	*A. hydrophila*	NZ_JEML00000000.1	72
259	Malaysia	non-vAh	Clinical	*A. hydrophila*	*A. dhakensis*	NZ_AOBP00000000.1	72
277	Malaysia	non-vAh	Clinical	*A. hydrophila*	*A. dhakensis*	NZ_AOBQ00000000.1	72
RB-AH	Canada	non-vAh	Soil	*A. hydrophila*	*A. hydrophila*	NZ_JPEH00000000.1	76
CIP 107985	Thailand	non-vAh	Frog	*A. hydrophila* subsp. *ranae*	*A. hydrophila*	NZ_CDDC00000000.1	77
YL17	Malaysia	non-vAh	Compost	*A. hydrophila*	*A. dhakensis*	NZ_CP007518.2	78
SSU	USA	non-vAh	Human	*A. hydrophila*	*A. dhakensis*	NZ_AGWR00000000.1	79
665N	Spain	non-vAh	Seafood	*A. bivalvium*	*A. bivalvium*	DQ504430	80
ESV-353	Mexico	non-vAh	Rainbow trout	*A. bestiarum*	*A. bestiarum*	KJ743516.1	71
ESV-364	Mexico	non-vAh	Rainbow trout	*A. bestiarum*	*A. bestiarum*	KJ743524.1	71
ESV-367	Mexico	non-vAh	Rainbow trout	*A. bestiarum*	*A. bestiarum*	KJ743526.1	71
0.2	Spain	non-vAh	Oscar	*A. caviae*	*A. caviae*	OP198648	This study
1P11S3	Malaysia	non-vAh	Basa fish	*A. dhakensis*	*A. dhakensis*	NZ_JADPIC000000000	81
KOR1	China (P.R.C.)	non-vAh	Mangrove	*A. dhakensis*	*A. dhakensis*	NZ_LJOE00000000.1	82
P1S3	Bangladesh	non-vAh	Pabda	*A. dhakensis*	*A. dhakensis*	OP198647	This study
P2L2	Bangladesh	non-vAh	Pabda	*A. dhakensis*	*A. dhakensis*	OP198646	This study
P3I3	Bangladesh	non-vAh	Pabda	*A. dhakensis*	*A. dhakensis*	OP222574	This study
P3L1	Bangladesh	non-vAh	Pabda	*A. dhakensis*	*A. dhakensis*	OP222573	This study
P3L2	Bangladesh	non-vAh	Pabda	*A. dhakensis*	*A. dhakensis*	OP222572	This study
P3L3	Bangladesh	non-vAh	Pabda	*A. dhakensis*	*A. dhakensis*	OP222571	This study
P3S1	Bangladesh	non-vAh	Pabda	*A. dhakensis*	*A. dhakensis*	OP222570	This study
P3S3	Bangladesh	non-vAh	Pabda	*A. dhakensis*	*A. dhakensis*	OP222569	This study
HE40	Finland	non-vAh	FW lake water	*A. finlandensis*	*A. finlandensis*	HG970924.1	83
4287D	Finland	non-vAh	FW lake water	*A. finlandensis*	*A. finlandensis*	NZ_JRGK00000000.1	29
4AK4	China	non-vAh	Carp	*A. hydrophila*	*A. media*	NZ_CP006579.1	84
ESV-360	Mexico	non-vAh	Rainbow trout	*A. media*	*A. media*	KJ743508.1	71
ESV-383	Mexico	non-vAh	Rainbow trout	*A. media*	*A. media*	KJ743513.1	71
R100	Spain	non-vAh	Trout	*A. media*	*A. hydrophila*	KP400944.1	85
AH31	Norway	non-vAh	Crab	*A. media*	*A. media*	KP400946.1	86
BWH65	USA	non-vAh	Perch	*A. caviae*	*A. media*	NZ_LESK00000000.1	12
0890	France	non-vAh	Koi carp	*A. media*	*A. media*	OP222568	This study
18900	USA	non-vAh	Canadian perch	*A. salmonicida*	*A. salmonicida*	JANLFD000000000	This study
ESV-355	Mexico	non-vAh	Rainbow trout	*A. sobria*	*A. sobria*	KJ743518.1	71
ESV-396	Mexico	non-vAh	Rainbow trout	*A. salmonicida*	*A. salmonicida*	KJ743550.1	71
ESV-400	Mexico	non-vAh	Rainbow trout	*A. veronii*	*A. veronii*	KJ743553.1	71
0.15	Spain	non-vAh	Oscar	*A. veronii*	*A. veronii*	OP222567	This study
D47366	Spain	non-vAh	Human feces	*A. veronii*	*A. veronii*	OP222566	This study
ESV-393	Mexico	non-vAh	Rainbow trout	*A. veronii*	*A. sobria*	KJ743548.1	71
ESV-397	Mexico	non-vAh	Rainbow trout	*A. veronii*	*A. veronii*	KJ743551.1	71
EN3600	China (P.R.C.)	non-vAh	Human tissue	*E. cloacae*	*E. cloacae*	NZ_CP035633.1	87
GGT036	Korea	non-vAh	Soil	*E. cloacae*	*E. cloacae*	NZ_CP009756.1	88
M12X01451	USA	non-vAh	Human tissue	*E. cloacae*	*E. cloacae*	NZ_CP017475.1	89
B1	Ghana	non-vAh	Tilapia	Plesiomonas shigelloides	P. shigelloides	OP222552	This study
D1	Ghana	non-vAh	Tilapia	*Enterobacter* spp.	*Enterobacter* spp.	OP222553	This study
E1	Ghana	non-vAh	Tilapia	P. shigelloides	P. shigelloides	OP222554	This study
G1	Ghana	non-vAh	Tilapia	P. shigelloides	P. shigelloides	OP222555	This study
B2	Ghana	non-vAh	Tilapia	*A. veronii*	*A. veronii*	OP222556	This study
F2	Ghana	non-vAh	Tilapia	P. shigelloides	P. shigelloides	OP222557	This study
8	Pakistan	non-vAh	Rohu	P. aeruginosa	P. aeruginosa	OP222558	This study
21	Pakistan	non-vAh	Rohu	P. aeruginosa	P. aeruginosa	OP222559	This study
37	Pakistan	non-vAh	Gulfam	P. aeruginosa	P. aeruginosa	OP222560	This study
38	Pakistan	non-vAh	Rohu	P. aeruginosa	P. aeruginosa	OP222561	This study
53A	Pakistan	non-vAh	Rainbow trout	Serratia liquefaciens	S. liquefaciens	OP222562	This study
62	Pakistan	non-vAh	Silver carp	P. aeruginosa	P. aeruginosa	OP222563	This study
63	Pakistan	non-vAh	Rohu	Eneterobacter cancerogenus	*E. cancerogenus*	OP222564	This study
PB	USA	non-vAh	Rainbow trout	*A. sobria*	*A. sobria*	OP222565	This study

**FIG 1:**
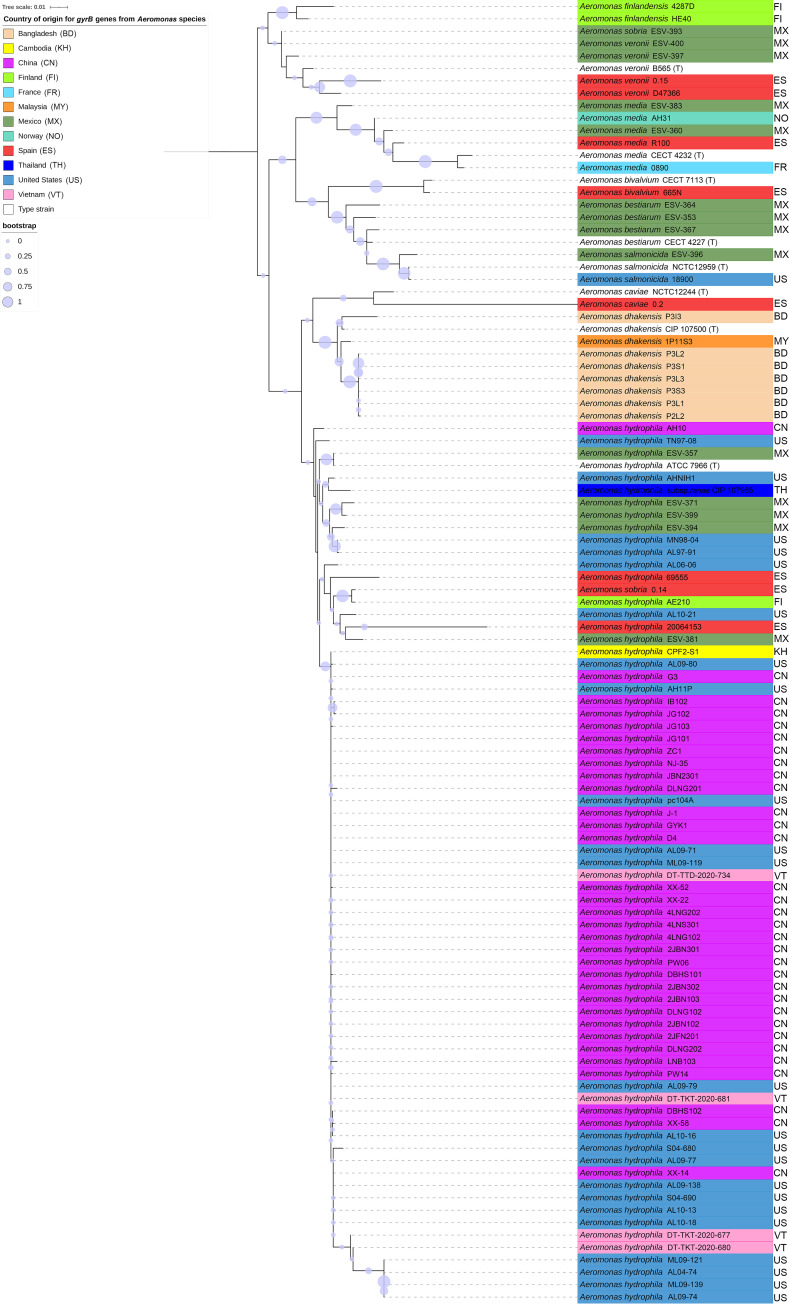
Phylogeny of *Aeromonas* species isolates based on the *gyrB* gene. The evolutionary relationships of vAh and other *Aeromonas* sp. isolates were inferred using the maximum likelihood method based on *gyrB* gene sequences. A total of 1,000 iterations were performed for determination of bootstrap support, with bootstrap values indicated by the size of the circle at each supported node.

The 43 *myo*-inositol-utilizing *Aeromonas* strains collected worldwide were subjected to vAh-specific and/or *gyrB*-targeted PCR using the primer sets listed in Table 2. A phylogenetic analysis was conducted using *gyrB* sequences from these isolates in addition to *Aeromonas* sp. type strains and previously described vAh strains from China and the United States (Fig. 1). The phylogeny of these strains revealed a great diversity of *Aeromonas* spp. that were obtained in this survey, including A. bestiarum, A. bivalvium, A. caviae, A. dhakensis, A. finlandensis, A. media, A. salmonicida, A. sobria, and A. veronii. Interestingly, some of these *Aeromonas* spp. had not been previously shown to have the ability to use *myo*-inositol as a carbon source, including *A. bestiarum*, *A. bivalvium*, *A. caviae*, *A. dhakensis*, *A. media*, and *A. veronii* (26, 28–30). While these *Aeromonas* species were not the target of this survey, this adds to our knowledge of the use of *myo-*inositol among diverse *Aeromonas* species. Additionally, it suggests that this ability may contribute to the persistence of these bacteria in aquatic habitats and the virulence of these opportunistic pathogens in diverse warm-water fish species.

**TABLE 2:** Primer sets for PCR to amplify *gyrB* or vAh-specific genetic loci^*a*^

Primer set	Direction	Sequence	Amplicon size (bp)
2986F	Forward	5′-CTATTACTGCCCCCTCGTTC-3′	167
2986R	Reverse	5′-ATTGAGCGGTATGCTGTCG-3′	
vAh-*SerF*	Forward	5′-AG′CATCACCAGCGTTGGCCC-3′	502
vAh-*SerR*	Reverse	5′-GCCGGGCTGAACTTCCGCAT-3′	
*gyrB*3F	Forward	5′-TCCGGCGGTCTGCACGGCGT-3′	680
*gyrB*9R	Reverse	5′-ACCTTGACGGAGATAACGGC-3′	
*gyrB*7F	Forward	5′-GGGGTCTACTGCTTCACCAA-3′	680
*gyrB*14R	Reverse	5′-TTGTCCGGGTTGTACTCGTC-3′	

a Primer sets 2986F/R and vAh-*Ser*F/R were used for vAh identification, and primer sets *gyrB*3F/9R and 7F/14R were used for *gyrB* amplification.

Based on the *gyrB* phylogeny, A. hydrophila strains isolated from Spain, Mexico, Finland, Cambodia, and Vietnam grouped together and formed well-supported clades. Furthermore, the *gyrB* phylogeny indicated that all previously described vAh strains (i.e., ST251) grouped together within a monophyletic clade with bootstrap support, clearly distinct from other *myo*-inositol utilizing *Aeromonas* sp. strains (Fig. 1). Interestingly, the vAh clade included strains from Cambodia (CPF2-S1) and Vietnam (DT-TKT-2020-677, DT-TKT-2020-680, DT-TKT-2020-681, and DT-TTD-2020-734). The Cambodian vAh strain CPF2-S1 was one of five *myo*-inositol utilizing bacterial isolates that were positive for vAh-specific PCR and isolated from Pangas catfish in the Mekong River basin. The four vAh isolates from Vietnam were all obtained from diseased striped catfish in the Mekong River delta, and three of them (DT-TKT-2020-677, DT-TKT-2020-680, DT-TKT-2020-681) are closely related to a recently reported vAh strain, DT-TTD-2020-734, obtained from striped catfish in the Mekong River delta (31). These newly described vAh strains indicate that additional fish species are susceptible to vAh and that the Mekong River basin is an active region of vAh disease transmission.

### Inositol catabolism phylogeny.

The evolutionary history of the inositol catabolism pathway among *myo*-inositol-utilizing *Aeromonas* spp. was inferred based on the amino acid sequences of IolA, IolC, IolD, IolE, IolG, InoE, InoF, and InoL, which were obtained from representative vAh and other *Aeromonas* sp. genomes (Fig. S1A to H). The variability in inositol gene content among these strains precluded a concatenated phylogenetic analysis. Among vAh strains, there were no differences observed in the evolutionary history of gene products required for *myo-*inositol transport (InoE, InoF, and InoL) or catabolism (IolA, IolC, IolD, IolE, and IolG), with all vAh strains present in the same monophyletic clade and having strong bootstrap support (Fig. S1A to H). This is consistent with the observation that vAh strains are clonal, including the newly isolated vAh strains from Cambodia (CPF2-S1) and Vietnam (e.g. DT-TTD-2020-734). Another consistent observation was that the inositol-related gene products from vAh strains share a close relationship with orthologous sequences from Enterobacter cloacae, which has been hypothesized to be the origin of the inositol catabolism pathway present in vAh strains (27). In contrast, the sequences obtained from other *Aeromonas* species were distantly related to those from vAh strains and E. cloacae, including *A. dhakensis* 1P11S3, *A. dhakensis* P3I3, *A. dhakensis* P3L3, *A. media* R100, *A. sobria* ESV-355, and *A. sobria* ESV-393. The evolutionary history of inositol utilization among *Aeromonas* sp. therefore appears to be complex, with horizontal gene transfer of inositol transport and catabolism postulated to play an important role. This survey revealed a large diversity of other *Aeromonas* species that can utilize *myo*-inositol. Future research should explore the role of *myo-*inositol utilization in the persistence and virulence of opportunistic *Aeromonas* sp. pathogens.

The role of *myo-*inositol utilization in vAh persistence and virulence should also be further explored. Channel catfish have been shown to synthesize *myo*-inositol in brain, kidney, and liver tissues, and soy-based fish feed containing a high concentration of phytic acid (inositol hexaphosphate) (32, 33). The inositol derived from fish tissues and dietary sources may provide both a carbon source and an environmental signal that induces expression of vAh virulence factors. The transcriptional regulator IolR is responsible for the regulation of *iol* genes as well as other virulence factors in bacterial pathogens, such as Salmonella enterica (34, 35). IolR has also been found to regulate autoaggregation and biofilm formation in the vAh strain NJ-35 (36). Furthermore, the presence of *myo*-inositol that accumulates in sediment from fish feed may help vAh to persist within the environment.

### Average nucleotide identity (ANI).

**FIG 2:**
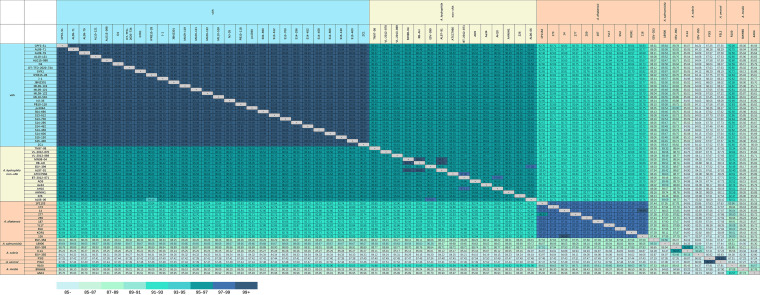
Pairwise comparison of average nucleotide identity (ANI) of vAh, non-vAh, and *myo*-inositol-utilizing *Aeromonas* sp. isolates. Genome sequences of vAh, non-vAh, and *myo*-inositol-utilizing *Aeromonas* sp. isolates were pairwise compared using JSpeciesWS. ANI values of >95% indicate that two strains belong to the same species.

The pairwise ANI comparisons for 63 *Aeromonas* sp. genomes, including representative vAh strains from China, Cambodia, the United States, and Vietnam showed high ANI values (>99%) for all vAh strains (Fig. 2), which was consistent with the previous core genome-based phylogeny indicating the clonality of all known vAh strains (12). In contrast, only a few non-vAh A. hydrophila strains showed high ANI values compared with vAh strains, and most ANI values ranged from 96% to 97%. The exceptions to this were strains that had been putatively indicated as *A. media*, *A. sobria*, and *A. veronii* based on phylogenetic analyses, all of which had discrepancies between the species affiliation indicated by ANI values and their species affiliation indicated in GenBank as previously described (37). Based on these ANI data and a core genome-based phylogeny, the phylogenetic affiliations of several strains were revised (see below and Table 1). This survey also included diverse *Aeromonas* sp. isolated from diseased fish and other environments, as revealed by the A. hydrophila-*Aeromonas* sp. pairwise ANI comparisons that ranged from 67% to 93%.

### *Aeromonas* core genome phylogenetic analysis.

**FIG 3:**
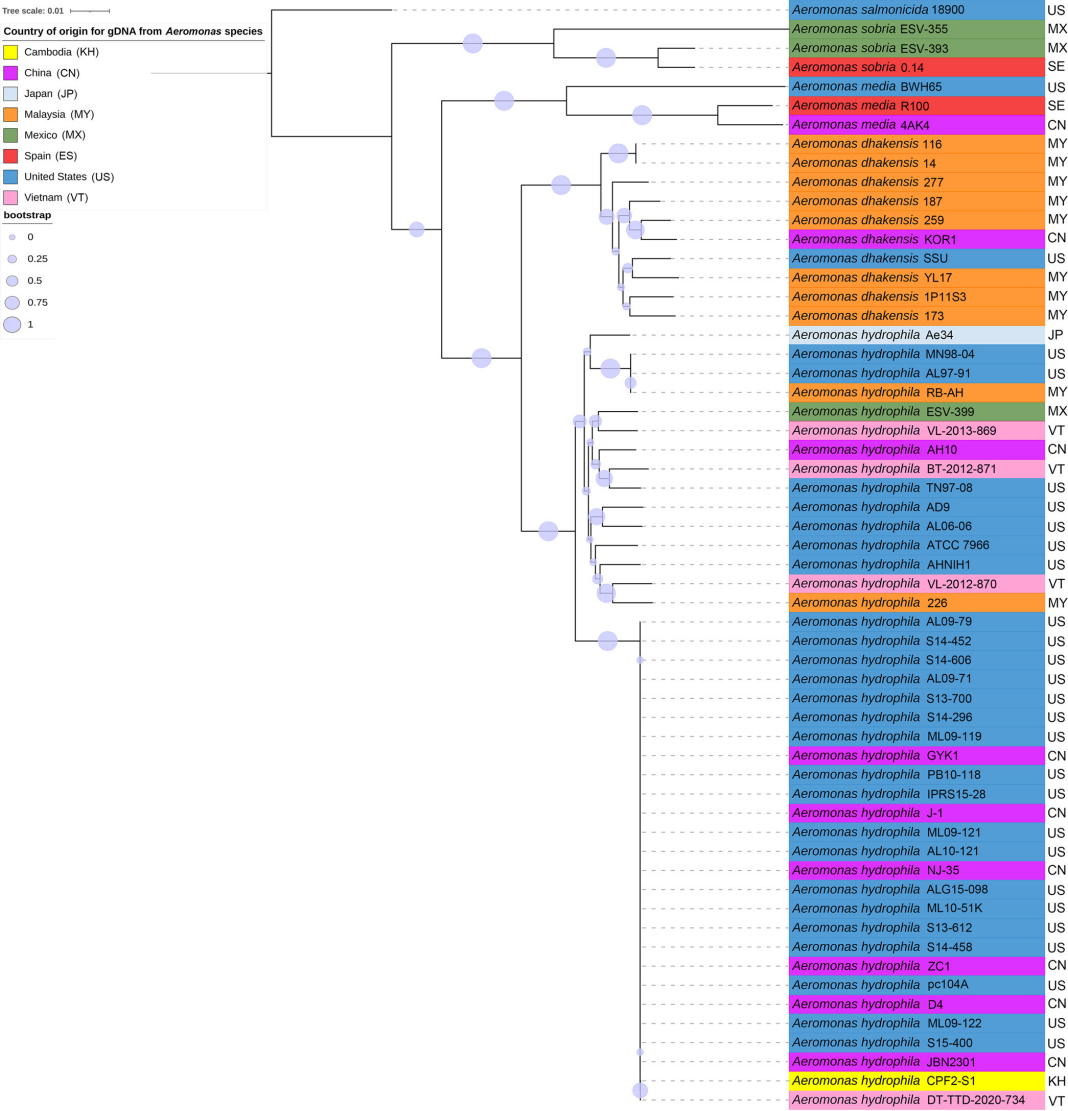
Phylogeny of *Aeromonas* sp. isolates based on the core genome sequences. The evolutionary relationships of vAh and other *Aeromonas* sp. isolates were inferred using the maximum likelihood method, based on core genome sequences. A total of 1,000 bootstrap replications were conducted, and bootstrap values are represented by the size of the circle for each supported node.

The phylogenetic relationships among the representative vAh and diverse *Aeromonas* sp. strains included in this survey were inferred based on a set of core genome sequences totaling 3.8 Mb (Fig. 3). A subset of vAh strains was included in the core genome phylogeny due to some of the strains lacking high-quality genome sequences (e.g., Vietnamese vAh strains DT-TKT-2020-681 DT-TKT-2020-677, DT-TKT-2020-680). Consistent with the *gyrB* phylogeny, the *Aeromonas* core genome phylogeny indicated that all vAh strains, including the newly identified strains from Cambodia and Vietnam, form a monophyletic clade with strong bootstrap support that is distinct from other A. hydrophila or other *Aeromonas* sp. strains (Fig. 3). While the clonal vAh clade showed little variation among its members for the core genome phylogeny, there was significant intraspecies genetic variability observed among the other *Aeromonas* sp. Strains, including within A. hydrophila, *A. dhakensis*, *A. media*, and *A. sobria*. Based on this core genome phylogeny (and ANI values), there were many bacterial isolates described as A. hydrophila that were affiliated with *A. dhakensis*, *A. media*, or *A. sobria*, and these revised phylogenetic affiliations have been indicated (Table 1). In this analysis, the exclusion of small fragments was set to 10 kbp because these fragments were found to be flanked by highly repetitive sequences, which were previously demonstrated to contribute less to the production of core genomes. This removal was chosen as a blanket approach to increase computational efficiency and decrease the noise generated from repetitive sequences, as this study is solely based on sequence-based comparisons. However, with the growing body of knowledge that shows repetitive regions as significant in regulation, future studies should focus on these noncoding regions.

### Virulence factors encoded in *Aeromonas* sp. genomes.

**FIG 4:**
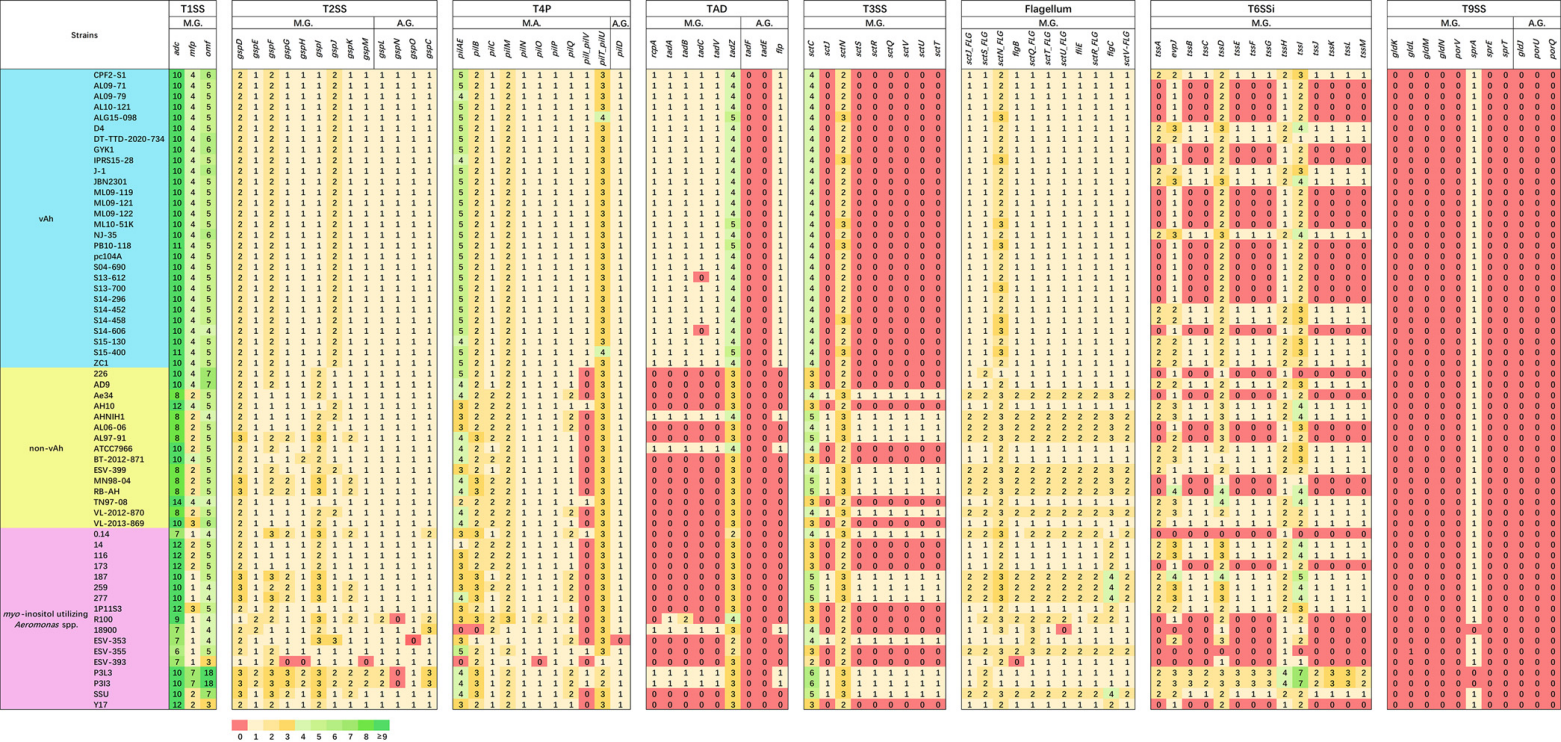
Predicted virulence factors for vAh and other *Aeromonas* sp. strains. *Aeromonas* genomes were annotated using RAST and submitted to MacSyFinder for secretion system analysis. Maximum independent E value and minimal profile coverage were set as the default, while the maximum E value was set as 1.0. Virulence factors include type 1 secretion system (T1SS), type 2 secretion system (T2SS), type 4 pili (T4P), tight adherence system (TAD), type 3 secretion system (T3SS), flagellum, a phylogenetic subtype of type 6 secretion system (T6SSi), and type 9 secretion system (T9SS).

Representative vAh and non-vAh genomes were evaluated for their encoded potential to secrete virulence factors (Fig. 4). In agreement with previous studies, vAh strains were universally found to encode complete type 2 secretion systems, which have been found to be essential to the virulence of a vAh strain isolated from a channel catfish in the United States (38). In contrast, type 3 secretion systems were only identified in non-vAh strains. Interestingly, the type 6 secretion systems (T6SS) were complete only in a subset of vAh strains as has been previously described (23). Most of the vAh isolates from China, with the one exception of strain GYK1, were predicted to possess the complete T6SS, which has been shown to contribute to biofilm formation and virulence in fish (24). The two new vAh isolates from Cambodia and Vietnam (CPF2-S1, DT-TTD-2020-734) possessed the entire T6SS, which further demonstrates their close relationship to vAh strains isolated from carp in China. In contrast, many of the vAh strains isolated from channel catfish in the United States lacked a complete T6SS, with the notable exception of S14-452 and other strains isolated from the Mississippi delta (23).

### VAh core genome phylogenetic analysis.

**FIG 5:**
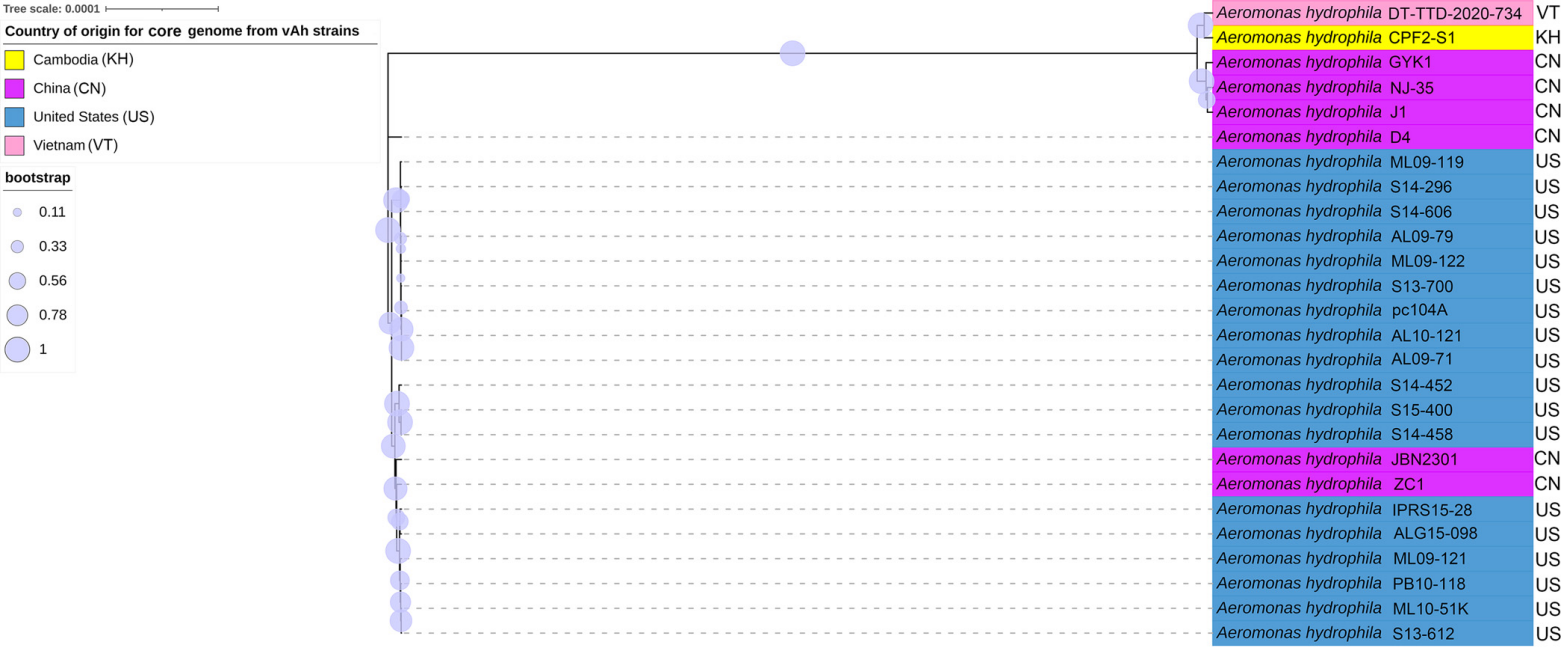
Phylogeny of vAh isolates based on the core genome sequences. The evolutionary relationships of vAh isolates were inferred using the maximum likelihood method, based on core genome sequences. A total of 1,000 bootstrap replications were conducted, and bootstrap values are represented by the size of the circle for each supported node.

The phylogenetic relationships among the representative vAh strains included in this survey were inferred based on a set of core genome sequences present in all sequenced vAh strains (Fig. 5). The vAh core genome phylogeny indicated that the newly identified strains obtained from diseased fish in Cambodia and Vietnam form a monophyletic clade with strong bootstrap support with vAh strains isolated from crucian carp (Carassius carassius) and mandarin fish (Siniperca chuatsi) in China. Moreover, these vAh isolates from Cambodia and Vietnam share a close relationship, indicating that they originated from a common ancestor. In contrast, two other strains isolated from carp in China, ZC1 and JBN2301, form a well-supported clade with vAh strains isolated from catfish in the United States (4).

The successful isolation of vAh from farmed Pangas catfish in Cambodia and from farmed striped catfish in Vietnam broadens the knowledge of the geographical distribution of vAh and the fish species in which this emerging pathogen can cause disease. Due to the rapid growth of the live fish trade in Asia and beyond, this pathogen could be transmitted to more countries and infect more fish species without sufficient biosafety (39). This calls for future development of rapid and inexpensive diagnostic assays to identify vAh strains and aid in biosecurity precautions to prevent further dissemination of this virulent pathogen.

## MATERIALS AND METHODS

### Bacterial isolates.

Fish that demonstrated the typical symptoms of MAS, especially with external hemorrhaging and in farms experiencing high fish mortality, were collected for diagnosis and autopsy at the local institution. *Aeromonas* sp. isolates were recovered from diseased fish from aquaculture ponds in Bangladesh, Cambodia, Finland, France, Ghana, Malaysia, Mexico, Norway, Pakistan, Spain, Thailand, and Vietnam (Table 1). The fish species sampled were tilapia (Oreochromis niloticus), striped catfish, pabda, Pangas catfish, basa catfish, rainbow trout, carp (*Cyprinidae* spp.), and perch (*Perca* spp.), while in some cases isolates were obtained from other environmental samples such as lake water, crab, seafood, and human feces (Table 1). Organs with the highest concentration of vAh, including liver, spleen, and kidney, were used to inoculate tryptic soy agar (TSA) plates (Beckton Dickinson, New Jersey, USA) or other bacteriologic growth medium appropriate for A. hydrophila cultivation, and these cultures were incubated at 30°C for 24 to 48 h. The vAh strain ML09-119 served as a control for comparison.

Single colonies that showed A. hydrophila morphology were cultured on TSA (30°C, 24 h) to obtain isolated colonies. Three colonies of each strain were cultured separately in 2 mL of M9 broth medium supplemented with 5.5 mM *myo*-inositol as previously described (27). The vAh strain ML09-119 and the non-vAh strain AL06-06 served as positive and negative controls, respectively. Cultures were grown at 30°C for 48 h to record their growth as measured by the optical density at 600 nm (OD_600_). The utilization of *myo*-inositol of an unknown isolate was monitored by turbidity and CFU counts (as previously described). An increase in turbidity (change in OD_600_ of >0.4) was observed for *myo*-inositol-utilizing strains over 48 h. Pure cultures of *myo*-inositol-utilizing strains were subsequently identified as vAh by phylogenetic analysis of *gyrB* sequences following previously described methods (12, 40). Validated vAh strains were cryopreserved in tryptic soy broth (TSB) containing 20% glycerol at −80°C.

### Phylogenetic analysis based on *gyrB* from *myo*-inositol-utilizing strains.

Genomic DNA of the *myo*-inositol-utilizing isolates was isolated using the E.Z.N.A. bacterial DNA isolation kit according to the manufacturer’s protocol (Omega Bio-Tek, Norcross, GA, USA). Bacterial DNA was quantified with a NanoDrop instrument (Thermo Fisher Scientific, Waltham, MA, USA) and used as a template for PCR amplify *gyrB* gene sequences using *Aeromonas* genus-level primer sets (Table 2) (41). To avoid the potential off-target priming and increase PCR specificity (42), touchdown PCR was conducted to generate *gyrB* products and performed on a Mastercycler Nexus thermo cycler (Eppendorf, Hamburg, Germany) with 50 ng of genomic DNA (gDNA) isolated from each strain, 25 μL of EconoTaq Plus green 2X master mix (Lucigen Corp., Middleton, WI, USA), and 0.5 μL of 20 μΜ reverse and forward primers. The thermal cycling parameters were 94°C for 3 min, 10 cycles of 94°C for 30 sec, 68°C for 30 sec (−1°C per cycle), and 72°C for 1 min, and then 25 cycles of 94°C for 30 sec, 58°C for 30 sec, and 72°C for 1 min and a final extension at 72°C for 5 min.

The *gyrB* gene amplicons were Sanger sequenced as described previously (27, 40) and assembled into consensus sequences using CLC Genomics Workbench (Qiagen, Inc., Aarhus, Denmark). The *gyrB* sequence reads were trimmed for quality, assembled into consensus sequences, and aligned with an existing *gyrB* sequence database obtained from Chinese and U.S. vAh and non-vAh strains (12), using ClustalW in MEGA X (43). The *gyrB* sequence database included sequences varying from 422 to 1,068 bp and included *Aeromonas* sp. type strains to confirm species affiliations. Phylogenetic relationships of the inositol**-**utilizing *Aeromonas* sp. strains and appropriate type strains were determined by the construction of a phylogenetic tree using MEGA X (43). In total, 100 strains were included in the tree, including 5 new vAh strains and 38 new non-vAh strains. Among the 66 A. hydrophila strains, some were removed due to poor sequence quality and/or the lack of an available viable culture from which to recover a better-quality *gyrB* sequence. The evolutionary history of the strains in the *gyrB* database was inferred using the maximum likelihood (ML) method (44). The ML analysis was conducted with 1,000 iterations for bootstrap support, with bootstrap values shown on each branch of the *gyrB* tree as a circle proportional to bootstrap support. The *gyrB* tree was annotated and visualized using iTOL v6 (45). The *gyrB* tree was rooted using the *A. sobria* type strain.

### Genome sequencing based on the *gyrB* phylogeny.

Representative isolates of different inositol-utilizing *Aeromonas* lineages were selected for Illumina sequencing based on the results of the *gyrB* phylogenetic tree. The sequenced strains were selected to represent vAh and inositol-utilizing *Aeromonas* from multiple geographical locations, including Spain, Mexico, Cambodia, Vietnam, Bangladesh, and Malaysia. The fragment libraries were constructed using a Nextera XT DNA library prep kit (Illumina, San Diego, CA, USA) based on the manufacturer’s protocol, followed by paired-end sequencing conducted on an Illumina MiSeq platform (46). Sequence reads were imported into CLC Genomics Workbench, which was used to trim sequences for quality, followed by *de novo* assembly using default settings. Draft genome contig sequences were generated for strains CPF2-S1 (Cambodia), 14 (Malaysia), 1P11S3 (Malaysia), ESV-393 (Mexico), ESV-399 (Mexico), R100 (Spain), VL-2013-869 (Vietnam), BT-2012-871 (Vietnam), VL-2012-870 (Vietnam), and DT-TTD-2020-734 (Vietnam). For subsequent phylogenomic analyses and prediction of virulence factors, a database was constructed that also included the existing sequences of vAh strains isolated from carp species in China and from catfish in the United States, along with other *Aeromonas* sp. strains isolated in China, the United States, Malaysia, and Japan.

### Inositol catabolism phylogeny.

A genomic database was generated that included draft genome sequences of strains isolated in Mexico, Spain, Cambodia, Bangladesh, Malaysia, and Vietnam that were supplemented with genome sequences of vAh strains from China and the United States, as well as from Enterobacter spp. which are predicted to be the origin of the inositol catabolism pathway present in vAh strains (22, 47). A total of 15 open reading frames (ORFs) were identified in the *myo*-inositol catabolism pathway in vAh strains, from which we selected proteins shown to be required for inositol utilization (IolA, IolC, IolD, IolE, and IolG) for phylogenetic analysis (48). The proteins predicted to be involved in *myo-*inositol transport, InoE, InoF, and InoL, were also included in the phylogenetic analyses (49). Given the unique evolutionary histories associated with these proteins involved in inositol catabolism, a separate phylogenetic analysis was conducted for each of these amino acid sequences. An ML tree with 1,000 iterations for bootstrap support was conducted on MEGA X as described above and visualized using ITOL v6.

### Average nucleotide identity.

To evaluate the overall genetic similarity between vAh, non-vAh, and *myo*-inositol-utilizing *Aeromonas* spp., the ANI values of the 63 *Aeromonas* genomes were compared using JSpeciesWS (50) and visualized with Daniel’s XL Toolbox v7.3.4 (51). According to the criteria for taxonomic affiliation of new genomes, an ANI value of >95% indicated that two strains belong to the same species (52, 53).

### *Aeromonas* core genome analysis.

Both noncoding and coding sequences of vAh and non-vAh strains, including *A. sobria*, *A. media*, *A. dhakensis*, and *A. salmonicida* strains, were used for a core genome phylogenetic analysis. In general, small fragments do not influence the overall quality of core genomes due to these small genomic regions being flanked by highly repetitive sequences. Removing small fragments helped to improve overall accuracy by decreasing the noise generated from repetitive sequences; therefore, any contigs less than 10 kb were filtered from *Aeromonas* genomes by limiting them from mapping to multiple regions. FASTA files of the filtered sequences were submitted to Mugsy v1.2.3, a multiple whole genome alignment tool, using default parameters (54). The alignment was processed by GBLOCK v0.91b for the identification of highly conserved regions across all *Aeromonas* spp. strains as previously described (55). The parameters used in GBLOCK for retention were dependent upon the input alignment as previously described (12). Briefly, a maximum of 8 contiguous regions, a minimum of 30 sequences for conserved regions, and 51 sequences for flanked positions were used for the gapped positions within a block. A ML tree based on the final alignment was generated using MEGAX with default parameters for the 60 *Aeromonas* spp. strains, including 28 vAh strains. The ML tree was further visualized using iTOL v6.

### VAh core genome phylogenetic analysis.

To assess the phylogeny of vAh strains isolated from Cambodia, China, the US, and Vietnam, a vAh core genome was created using both coding and noncoding sequences of representative vAh. Contigs less than 10 kb in size were removed to increase computational efficiency, and filtered data were submitted as FASTA files to the multiple whole-genome alignment tool Mugsy v1.2.3 (54), under default parameters. The resulting alignment was subsequently processed with GBLOCK v0.91b (55) in order to identify regions of high conservation across all isolates. Parameters for retention by GBLOCK are dictated by the input alignment and were the following: a minimum of 31 and 51 sequences for conserved and flanked positions, respectively, a maximum of 8 contiguous, but nonconserved positions, a minimum block length of 10, and one-half of the sequences allowed to possess gapped positions within a block. From the final alignment, a maximum likelihood (ML) phylogeny for the vAh isolates was inferred using RAxML v8.2.8 (56) under the general time reversible model of evolution with estimated proportions of invariable sites and rate variation among sites (i.e., GTR + I + G) and 1,000 bootstraps to determine branch supports, as described previously (22). Trees were visualized using iTOL v6.

### Virulence factor prediction.

Virulence factor prediction and identification of secretion systems followed previously described methods (23, 57). Briefly, the secretion systems of *Aeromonas* strains were identified with the program MacSyFinder. The data set option was set as “unordered” to evaluate the draft genome of each strain. The minimal profile coverage was set to 0.5, the maximum E value was set to 1.0, and the maximum independent E value was 0.001. Secretion systems of vAh, non-vAh, and *myo*-inositol-utilizing *Aeromonas* strains were identified and indicated with mandatory and accessory genes, and the corresponding copy numbers were determined.

### Data availability.

The sequences were deposited in the GenBank database under accession no. OP198646 to OP198653 for the *gyrB* sequences, and the genome sequences were deposited as JANLOJ000000000.1, NZ_JALRNI010000001.1, NZ_JALRNJ000000000.1, NZ_JALRNL000000000.1, NZ_JALRNK000000000.1, JANLFC000000000.1, and JANLFD000000000.1 (Table 1).

## References

[1] Isonhood JH, Drake M. 2002. Aeromonas species in foods. J Food Prot 65:575–582. doi:10.4315/0362-028x-65.3.575. 11899061

[2] Palumbo S, Williams A, Buchanan R, Phillips J. 1987. Thermal resistance of Aeromonas hydrophila. J Food Prot 50:761–764. doi:10.4315/0362-028X-50.9.761. 30978800

[3] Huys G, Pearson M, Kämpfer P, Denys R, Cnockaert M, Inglis V, Swings J. 2003. Aeromonas hydrophila subsp. ranae subsp. nov., isolated from septicaemic farmed frogs in Thailand. Int J Syst Evol Microbiol 53:885–891. doi:10.1099/ijs.0.02357-0. 12807217

[4] Hossain MJ, Sun D, McGarey DJ, Wrenn S, Alexander LM, Martino ME, Xing Y, Terhune JS, Liles MR. 2014. An Asian origin of virulent Aeromonas hydrophila responsible for disease epidemics in United States-farmed catfish. mBio 5:e00848-14. doi:10.1128/mBio.00848-14. 24895303 PMC4049099

[5] Liu J, Xie L, Zhao D, Yang T, Hu Y, Sun Z, Yu X. 2019. A fatal diarrhoea outbreak in farm-raised Deinagkistrodon acutus in China is newly linked to potentially zoonotic Aeromonas hydrophila. Transbound Emerg Dis 66:287–298. doi:10.1111/tbed.13020. 30222905

[6] Guerra IM, Fadanelli R, Figueiró M, Schreiner F, Delamare APL, Wollheim C, Costa SOP, Echeverrigaray S. 2007. Aeromonas associated diarrhoeal disease in south Brazil: prevalence, virulence factors and antimicrobial resistance. Braz J Microbiol 38:638–643. doi:10.1590/S1517-83822007000400011.

[7] Grizzle JM, Kiryu Y. 1993. Histopathology of gill, liver, and pancreas, and serum enzyme levels of channel catfish infected with Aeromonas hydrophila complex. J Aquat Anim Health 5:36–50. doi:10.1577/1548-8667(1993)005<0036:HOGLAP>2.3.CO;2.

[8] El-Son MA, Nofal MI, Abdel-Latif HM. 2021. Co-infection of Aeromonas hydrophila and Vibrio parahaemolyticus isolated from diseased farmed striped mullet (Mugil cephalus) in Manzala, Egypt: a case report. Aquaculture 530:735738. doi:10.1016/j.aquaculture.2020.735738.

[9] Plumb JA, Hanson LA. 2010. Health maintenance and principal microbial diseases of cultured fishes. John Wiley & Sons, New York, NY.

[10] Sakazaki R, Shimada T. 1984. O-serogrouping scheme for mesophilic Aeromonas strains. Jpn J Med Sci Biol 37:247–255. doi:10.7883/yoken1952.37.247. 6536785

[11] Wise AL, LaFrentz BR, Kelly AM, Khoo LH, Xu T, Liles MR, Bruce TJ. 2021. A review of bacterial co-infections in farmed catfish: components, diagnostics, and treatment directions. Animals 11:3240. doi:10.3390/ani11113240. 34827972 PMC8614398

[12] Rasmussen-Ivey CR, Hossain MJ, Odom SE, Terhune JS, Hemstreet WG, Shoemaker CA, Zhang D, Xu D-H, Griffin MJ, Liu Y-J, Figueras MJ, Santos SR, Newton JC, Liles MR. 2016. Classification of a hypervirulent Aeromonas hydrophila pathotype responsible for epidemic outbreaks in warm-water fishes. Front Microbiol 7:1615. doi:10.3389/fmicb.2016.01615. 27803692 PMC5067525

[13] Rasmussen-Ivey CR, Figueras MJ, McGarey D, Liles MR. 2016. Virulence factors of Aeromonas hydrophila: in the wake of reclassification. Front Microbiol 7:1337. doi:10.3389/fmicb.2016.01337. 27610107 PMC4997093

[14] Huaiqing C, Chengping L. 1991. Study on the pathogen of epidemic septicemia occurred in cultured cyprinoid fishes in southeastern China. Biodivers Sci 6:31–36.

[15] Deng G, Jiang X, Ye X, Liu M, Xu S, Liu L, Bai Y, Luo X. 2009. Isolation, identification and characterization of Aeromonas hydrophila from hemorrhagic grass carp. Microbiol China 36:1170–1177.

[16] Pang M, Jiang J, Xie X, Wu Y, Dong Y, Kwok AHY, Zhang W, Yao H, Lu C, Leung FC, Liu Y. 2015. Novel insights into the pathogenicity of epidemic Aeromonas hydrophila ST251 clones from comparative genomics. Sci Rep 5:9833. doi:10.1038/srep09833. 26014286 PMC4444815

[17] Zhang Y, Arakawa E, Leung K. 2002. Novel Aeromonas hydrophila PPD134/91 genes involved in O-antigen and capsule biosynthesis. Infect Immun 70:2326–2335. doi:10.1128/IAI.70.5.2326-2335.2002. 11953367 PMC127894

[18] Hemstreet B. 2010. An update on Aeromonas hydrophila from a fish health specialist for summer 2010. Catfish J 24:4.

[19] Hanson L, Liles M, Hossain M, Griffin M, Hemstreet W. 2014. Motile aeromonas septicemia. *In* Fish health section blue book. American Fisheries Society, Bethesda, MD.

[20] Changnon S. 2019. The great flood of 1993: causes, impacts, and responses. Routledge, New York, NY.

[21] Simoes AJG, Hidalgo CA. The economic complexity observatory: an analytical tool for understanding the dynamics of economic development. In Workshops at the twenty-fifth AAAI conference on artificial intelligence, San Francisco, CA.

[22] Wang N, Liu J, Pang M, Wu Y, Awan F, Liles MR, Lu C, Liu Y. 2018. Diverse roles of Hcp family proteins in the environmental fitness and pathogenicity of Aeromonas hydrophila Chinese epidemic strain NJ-35. Appl Microbiol Biotechnol 102:7083–7095. doi:10.1007/s00253-018-9116-0. 29862449

[23] Tekedar HC, Abdelhamed H, Kumru S, Blom J, Karsi A, Lawrence ML. 2018. Comparative genomics of Aeromonas hydrophila secretion systems and mutational analysis of hcp1 and vgrG1 genes from T6SS. Front Microbiol 9:3216. doi:10.3389/fmicb.2018.03216. 30687246 PMC6333679

[24] Ma S, Dong Y, Wang N, Liu J, Lu C, Liu Y. 2020. Identification of a new effector-immunity pair of Aeromonas hydrophila type VI secretion system. Vet Res 51:71. doi:10.1186/s13567-020-00794-w. 32448355 PMC7245790

[25] Al-Fatlawy HNK, Al-Hadrawy HA. 2014. Isolation and characterization of A. hydrophila from the Al-Jadryia River in Baghdad (Iraq). Am J Educ Res 2:658–662. doi:10.12691/education-2-8-14.

[26] Azzam-Sayuti M, Ina-Salwany MY, Zamri-Saad M, Annas S, Yusof MT, Monir MS, Mohamad A, Muhamad-Sofie MHN, Lee JY, Chin YK, Amir-Danial Z, Asyiqin A, Lukman B, Liles MR, Amal MNA. 2021. Comparative pathogenicity of Aeromonas spp. in cultured red hybrid tilapia (Oreochromis niloticus × O. mossambicus). Biology 10:1192. doi:10.3390/biology10111192. 34827185 PMC8614744

[27] Hossain MJ, Waldbieser GC, Sun D, Capps NK, Hemstreet WB, Carlisle K, Griffin MJ, Khoo L, Goodwin AE, Sonstegard TS, Schroeder S, Hayden K, Newton JC, Terhune JS, Liles MR. 2013. Implication of lateral genetic transfer in the emergence of Aeromonas hydrophila isolates of epidemic outbreaks in channel catfish. PLoS One 8:e80943. doi:10.1371/journal.pone.0080943. 24278351 PMC3835674

[28] von Graevenitz A, Bucher C. 1983. Evaluation of differential and selective media for isolation of Aeromonas and Plesiomonas spp. from human feces. J Clin Microbiol 17:16–21. doi:10.1128/jcm.17.1.16-21.1983. 6826700 PMC272566

[29] Beaz-Hidalgo R, Latif-Eugenín F, Hossain M, Berg K, Niemi R, Rapala J, Lyra C, Liles M, Figueras M. 2015. Aeromonas aquatica sp. nov., Aeromonas finlandiensis sp. nov. and Aeromonas lacus sp. nov. isolated from Finnish waters associated with cyanobacterial blooms. Syst Appl Microbiol 38:161–168. doi:10.1016/j.syapm.2015.02.005. 25852023

[30] Dallaire-Dufresne S, Barbeau X, Sarty D, Tanaka KH, Denoncourt AM, Lagüe P, Reith ME, Charette SJ. 2013. Aeromonas salmonicida Ati2 is an effector protein of the type three secretion system. Microbiology (Reading) 159:1937–1945. doi:10.1099/mic.0.067959-0. 23832001

[31] Ngo TP, Vu HT, Le TT, Bui HC, Liles MR, Rodkhum C. 2022. Comparative genomic analysis of hypervirulent Aeromonas hydrophila strains from striped catfish (Pangasianodon hypophthalmus) in Vietnam. Aquaculture 558:738364. doi:10.1016/j.aquaculture.2022.738364.

[32] Burtle G, Lovell R. 1989. Lack of response of channel catfish (Ictalurus punctatus) to dietary myo-inositol. Can J Fish Aquat Sci 46:218–222. doi:10.1139/f89-030.

[33] Jackson LS, Li MH, Robinson EH. 1996. Use of microbial phytase in channel catfish Ictalurus punctatus diets to improve utilization of phytate phosphorus 1. J World Aquacult Soc 27:309–313. doi:10.1111/j.1749-7345.1996.tb00613.x.

[34] Zhang D, Pridgeon JW, Klesius PH. 2013. Expression and activity of recombinant proaerolysin derived from Aeromonas hydrophila cultured from diseased channel catfish. Vet Microbiol 165:478–482. doi:10.1016/j.vetmic.2013.04.023. 23680108

[35] Cordero-Alba M, Bernal-Bayard J, Ramos-Morales F. 2012. SrfJ, a Salmonella type III secretion system effector regulated by PhoP, RcsB, and IolR. J Bacteriol 194:4226–4236. doi:10.1128/JB.00173-12. 22661691 PMC3416237

[36] Dong Y, Li S, Zhao D, Liu J, Ma S, Geng J, Lu C, Liu Y. 2020. IolR, a negative regulator of the myo-inositol metabolic pathway, inhibits cell autoaggregation and biofilm formation by downregulating RpmA in Aeromonas hydrophila. NPJ Biofilms Microbiomes 6:1–12. doi:10.1038/s41522-020-0132-3. 32433466 PMC7239862

[37] Beaz-Hidalgo R, Hossain MJ, Liles MR, Figueras M-J. 2015. Strategies to avoid wrongly labelled genomes using as example the detected wrong taxonomic affiliation for Aeromonas genomes in the GenBank database. PLoS One 10:e0115813. doi:10.1371/journal.pone.0115813. 25607802 PMC4301921

[38] Barger PC, Liles MR, Newton JC. 2020. Type II secretion is essential for virulence of the emerging fish pathogen, hypervirulent Aeromonas hydrophila. Front Vet Sci 7:574113. doi:10.3389/fvets.2020.574113. 33088835 PMC7544816

[39] Kobayashi M, Msangi S, Batka M, Vannuccini S, Dey MM, Anderson JL. 2015. Fish to 2030: the role and opportunity for aquaculture. Aquacult Econ Manage 19:282–300. doi:10.1080/13657305.2015.994240.

[40] Griffin MJ, Goodwin AE, Merry GE, Liles MR, Williams MA, Ware C, Waldbieser GC. 2013. Rapid quantitative detection of Aeromonas hydrophila strains associated with disease outbreaks in catfish aquaculture. J Vet Diagn Invest 25:473–481. doi:10.1177/1040638713494210. 23847227

[41] Yanez M, Catalán V, Apraiz D, Figueras M, Martinez-Murcia A. 2003. Phylogenetic analysis of members of the genus Aeromonas based on gyrB gene sequences. Int J Syst Evol Microbiol 53:875–883. doi:10.1099/ijs.0.02443-0. 12807216

[42] Green MR, Sambrook J. 2018. Touchdown polymerase chain reaction (PCR). Cold Spring Harb Protoc. doi:10.1101/pdb.prot095133. 29717053

[43] Kumar S, Stecher G, Li M, Knyaz C, Tamura K. 2018. MEGA X: molecular evolutionary genetics analysis across computing platforms. Mol Biol Evol 35:1547–1549. doi:10.1093/molbev/msy096. 29722887 PMC5967553

[44] Guindon S, Dufayard J-F, Lefort V, Anisimova M, Hordijk W, Gascuel O. 2010. New algorithms and methods to estimate maximum-likelihood phylogenies: assessing the performance of PhyML 3.0. Syst Biol 59:307–321. doi:10.1093/sysbio/syq010. 20525638

[45] Letunic I, Bork P. 2021. Interactive Tree of Life (iTOL) v5: an online tool for phylogenetic tree display and annotation. Nucleic Acids Res 49:W293–W296. doi:10.1093/nar/gkab301. 33885785 PMC8265157

[46] Sato MP, Ogura Y, Nakamura K, Nishida R, Gotoh Y, Hayashi M, Hisatsune J, Sugai M, Takehiko I, Hayashi T. 2019. Comparison of the sequencing bias of currently available library preparation kits for Illumina sequencing of bacterial genomes and metagenomes. DNA Res 26:391–398. doi:10.1093/dnares/dsz017. 31364694 PMC6796507

[47] Hossain M. 2012. Molecular Interactions between phage and the catfish pathogen Edwardsiella ictaluri and comparative genomics of epidemic strains of Aeromonas hydrophila. PhD thesis. Auburn University, Auburn, AL.

[48] Yoshida K, Yamaguchi M, Morinaga T, Kinehara M, Ikeuchi M, Ashida H, Fujita Y. 2008. myo-Inositol catabolism in Bacillus subtilis. J Biol Chem 283:10415–10424. doi:10.1074/jbc.M708043200. 18310071

[49] Rodionova IA, Leyn SA, Burkart MD, Boucher N, Noll KM, Osterman AL, Rodionov DA. 2013. Novel inositol catabolic pathway in Thermotoga maritima. Environ Microbiol 15:2254–2266. doi:10.1111/1462-2920.12096. 23441918

[50] Richter M, Rosselló-Móra R, Oliver Glöckner F, Peplies J. 2016. JSpeciesWS: a web server for prokaryotic species circumscription based on pairwise genome comparison. Bioinformatics 32:929–931. doi:10.1093/bioinformatics/btv681. 26576653 PMC5939971

[51] Kraus D. 2014. Consolidated data analysis and presentation using an open-source add-in for the Microsoft Excel spreadsheet software. Med Writing 23:25–28. doi:10.1179/2047480613Z.000000000181.

[52] Figueras MJ, Beaz-Hidalgo R, Hossain MJ, Liles MR. 2014. Taxonomic affiliation of new genomes should be verified using average nucleotide identity and multilocus phylogenetic analysis. Genome Announcements 2:e00927-14. doi:10.1128/genomeA.00927-14. 25477398 PMC4256179

[53] Konstantinidis KT, Tiedje JM. 2005. Genomic insights that advance the species definition for prokaryotes. Proc Natl Acad Sci USA 102:2567–2572. doi:10.1073/pnas.0409727102. 15701695 PMC549018

[54] Angiuoli SV, Salzberg SL. 2011. Mugsy: fast multiple alignment of closely related whole genomes. Bioinformatics 27:334–342. doi:10.1093/bioinformatics/btq665. 21148543 PMC3031037

[55] Castresana J. 2000. Selection of conserved blocks from multiple alignments for their use in phylogenetic analysis. Mol Biol Evol 17:540–552. doi:10.1093/oxfordjournals.molbev.a026334. 10742046

[56] Stamatakis A. 2014. RAxML version 8: a tool for phylogenetic analysis and post-analysis of large phylogenies. Bioinformatics 30:1312–1313. doi:10.1093/bioinformatics/btu033. 24451623 PMC3998144

[57] Abby SS, Néron B, Ménager H, Touchon M, Rocha EP. 2014. MacSyFinder: a program to mine genomes for molecular systems with an application to CRISPR-Cas systems. PLoS One 9:e110726. doi:10.1371/journal.pone.0110726. 25330359 PMC4201578

[58] Pridgeon JW, Zhang D, Zhang L. 2014. Complete genome sequence of a moderately virulent Aeromonas hydrophila strain, pc104A, isolated from soil of a catfish pond in West Alabama. Genome Announc 2:e00554-14. doi:10.1128/genomeA.00554-14. 24903879 PMC4047458

[59] Yang W, Li N, Li M, Zhang D, An G. 2016. Complete genome sequence of fish pathogen Aeromonas hydrophila JBN2301. Genome Announc 4:e01615-15. doi:10.1128/genomeA.01615-15. 26823580 PMC4732333

[60] Liles M, Hemstreet W, Waldbieser G, Griffin M, Khoo L, Bebak J, Garcia J, Goodwin A, Capps N, Hayden K. 2011. Comparative genomics of Aeromonas hydrophila isolates from an epidemic in channel catfish. Poster 1489. American Society for Microbiology Meeting.

[61] Pan H, Wu S, Dong C, Shi C, Ye M, Lin T, Huang Z. 2004. Identification, virulence, hemolytic activity of GYK1, a strain of pathogenic Aeromonas hydrophila isolated from mandarinfish. J Shanghai Fish Univ 13:e29.

[62] Zhu L, Zheng J-S, Wang W-M, Luo Y. 2019. Complete genome sequence of highly virulent Aeromonas hydrophila strain D4, isolated from a diseased blunt-snout bream in China. Microbiol Resour Announc 8:e01035-18. doi:10.1128/MRA.01035-18. 30701228 PMC6346177

[63] Tekedar HC, Kumru S, Karsi A, Waldbieser GC, Sonstegard T, Schroeder SG, Liles MR, Griffin MJ, Lawrence ML. 2016. Draft genome sequences of four virulent Aeromonas hydrophila strains from catfish aquaculture. Genome Announcements 4:e00860-16. doi:10.1128/genomeA.00860-16. 27540076 PMC4991721

[64] Zhang Y, Gao X, Ye J, Chen N, Zhang X, Bing X. 2017. Molecular characterization and establishment of LAMP detection method of pathogenic Aeromonas hydrophila isolated from Siniperca chuatsi. Acta Hydrobiologica Sinica 41:1225–1231.

[65] Pridgeon J, Yildirim-Aksoy M, Klesius P, Kojima K, Mobley J, Srivastava K, Reddy P. 2013. Identification of gyrB and rpoB gene mutations and differentially expressed proteins between a novobiocin-resistant Aeromonas hydrophila catfish vaccine strain and its virulent parent strain. Vet Microbiol 166:624–630. doi:10.1016/j.vetmic.2013.07.025. 23968889

[66] Zhang X, Yang W, Li T, Li A. 2013. The genetic diversity and virulence characteristics of Aeromonas hydrophila isolated from fishponds with disease outbreaks in Hubei province. Acta Hydrobiologica Sinica 37:458–466.

[67] Hu M, Wang N, Pan Z, Lu C, Liu Y. 2012. Identity and virulence properties of Aeromonas isolates from diseased fish, healthy controls and water environment in China. Lett Appl Microbiol 55:224–233. doi:10.1111/j.1472-765X.2012.03281.x. 22725694

[68] Jagoda SDS, Tan E, Arulkanthan A, Kinoshita S, Watabe S, Asakawa S. 2014. Draft genome sequence of Aeromonas hydrophila strain Ae34, isolated from a septicemic and moribund koi carp (Cyprinus carpio koi), a freshwater aquarium fish. Genome Announcements 2:e00572-14. doi:10.1128/genomeA.00572-14. 24926056 PMC4056299

[69] Lenneman EM, Barney BM. 2014. Draft genome sequences of the alga-degrading bacteria Aeromonas hydrophila strain AD9 and Pseudomonas pseudoalcaligenes strain AD6. Genome Announcements 2:e00709-14. doi:10.1128/genomeA.00709-14. 25035334 PMC4102871

[70] Seshadri R, Joseph SW, Chopra AK, Sha J, Shaw J, Graf J, Haft D, Wu M, Ren Q, Rosovitz MJ, Madupu R, Tallon L, Kim M, Jin S, Vuong H, Stine OC, Ali A, Horneman AJ, Heidelberg JF. 2006. Genome sequence of Aeromonas hydrophila ATCC 7966T: jack of all trades. J Bacteriol 188:8272–8282. doi:10.1128/JB.00621-06. 16980456 PMC1698176

[71] Vega-Sánchez V, Acosta-Dibarrat J, Vega-Castillo F, Castro-Escarpulli G, Aguilera-Arreola MG, Soriano-Vargas E. 2014. Phenotypical characteristics, genetic identification, and antimicrobial sensitivity of Aeromonas species isolated from farmed rainbow trout (Onchorynchus mykiss) in Mexico. Acta Trop 130:76–79. doi:10.1016/j.actatropica.2013.10.021. 24211839

[72] Chan K-G, Puthucheary SD, Chan X-Y, Yin W-F, Wong C-S, Too W-SS, Chua K-H. 2011. Quorum sensing in Aeromonas species isolated from patients in Malaysia. Curr Microbiol 62:167–172. doi:10.1007/s00284-010-9689-z. 20544198

[73] Berg KA, Lyra C, Sivonen K, Paulin L, Suomalainen S, Tuomi P, Rapala J. 2009. High diversity of cultivable heterotrophic bacteria in association with cyanobacterial water blooms. ISME J 3:314–325. doi:10.1038/ismej.2008.110. 19020559

[74] Xu L, Wang H, Yang X, Lu L. 2013. Integrated pharmacokinetics/pharmacodynamics parameters-based dosing guidelines of enrofloxacin in grass carp Ctenopharyngodon idella to minimize selection of drug resistance. BMC Veterinary Res 9:126. doi:10.1186/1746-6148-9-126. PMC3717066 23800340

[75] Hughes HY, Conlan SP, Lau AF, Dekker JP, Michelin AV, Youn J-H, Henderson DK, Frank KM, Segre JA, Palmore TN. 2016. Detection and whole-genome sequencing of carbapenemase-producing Aeromonas hydrophila isolates from routine perirectal surveillance culture. J Clin Microbiol 54:1167–1170. doi:10.1128/JCM.03229-15. 26888898 PMC4809936

[76] Emond-Rheault J-G, Vincent AT, Trudel MV, Brochu F, Boyle B, Tanaka KH, Attéré SA, Jubinville É, Loch TP, Winters AD, Faisal M, Frenette M, Derome N, Charette SJ. 2015. Variants of a genomic island in Aeromonas salmonicida subsp. salmonicida link isolates with their geographical origins. Vet Microbiol 175:68–76. doi:10.1016/j.vetmic.2014.11.014. 25480167

[77] Colston SM, Fullmer MS, Beka L, Lamy B, Gogarten JP, Graf J. 2014. Bioinformatic genome comparisons for taxonomic and phylogenetic assignments using Aeromonas as a test case. mBio 5:e02136-14. doi:10.1128/mBio.02136-14. 25406383 PMC4251997

[78] Lim Y-L, Roberts RJ, Ee R, Yin W-F, Chan K-G. 2016. Complete genome sequence and methylome analysis of Aeromonas hydrophila strain YL17, isolated from a compost pile. Genome Announcements 4:e00060-16. doi:10.1128/genomeA.00060-16. 26941143 PMC4777754

[79] Erova TE, Sha J, Horneman AJ, Borchardt MA, Khajanchi BK, Fadl AA, Chopra AK. 2007. Identification of a new hemolysin from diarrheal isolate SSU of Aeromonas hydrophila. FEMS Microbiol Lett 275:301–311. doi:10.1111/j.1574-6968.2007.00895.x. 17725618

[80] Minana-Galbis D, Farfan M, Fusté MC, Lorén JG. 2007. Aeromonas bivalvium sp. nov., isolated from bivalve molluscs. Int J Syst Evol Microbiol 57:582–587. doi:10.1099/ijs.0.64497-0. 17329789

[81] Azzam-Sayuti M, Ina-Salwany MY, Zamri-Saad M, Annas S, Liles MR, Xu T, Amal MNA, Yusof MT. 2022. Draft genome sequence of myo-inositol utilizing Aeromonas dhakensis 1P11S3 isolated from striped catfish (Pangasianodon hypopthalmus) in a local fish farm in Malaysia. Data in Brief 41:107974. doi:10.1016/j.dib.2022.107974. 35252492 PMC8889345

[82] Yin M, Ma Z, Cai Z, Lin G, Zhou J. 2015. Genome sequence analysis reveals evidence of quorum-sensing genes present in Aeromonas hydrophila strain KOR1, isolated from a mangrove plant (Kandelia obovata). Genome Announcements 3:e01461-15. doi:10.1128/genomeA.01461-15. PMC4675955 26659690

[83] Berg A, Rødseth OM, Hansen T. 2007. Fish size at vaccination influence the development of side-effects in Atlantic salmon (Salmo salar L.). Aquaculture 265:9–15. doi:10.1016/j.aquaculture.2007.02.014.

[84] Gao X, Jian J, Li W-J, Yang Y-C, Shen X-W, Sun Z-R, Wu Q, Chen G-Q. 2013. Genomic study of polyhydroxyalkanoates producing Aeromonas hydrophila 4AK4. Appl Microbiol Biotechnol 97:9099–9109. doi:10.1007/s00253-013-5189-y. 24000047

[85] Talagrand-Reboul E, Roger F, Kimper J-L, Colston SM, Graf J, Latif-Eugenín F, Figueras MJ, Petit F, Marchandin H, Jumas-Bilak E, Lamy B. 2017. Delineation of taxonomic species within complex of species: Aeromonas media and related species as a test case. Front Microbiol 8:621. doi:10.3389/fmicb.2017.00621. 28458658 PMC5394120

[86] Granum PE, O’Sullivan K, Tomás JM, Ørmen Ø. 1998. Possible virulence factors of Aeromonas spp. from food and water. FEMS Immunol Med Microbiol 21:131–137. doi:10.1111/j.1574-695X.1998.tb01158.x. 9685002

[87] Yuan S, Wu G, Zheng B. 2019. Complete genome sequence of an IMP-8, CTX-M-14, CTX-M-3 and QnrS1 co-producing Enterobacter asburiae isolate from a patient with wound infection. J Glob Antimicrob Resist 18:52–54. doi:10.1016/j.jgar.2019.05.029. 31181270

[88] Gong G, Um Y, Park TH, Woo HM. 2015. Complete genome sequence of Enterobacter cloacae GGT036: A furfural tolerant soil bacterium. J Biotechnol 193:43–44. doi:10.1016/j.jbiotec.2014.11.012. 25444880

[89] Carter MQ, Pham A, Huynh S, He X. 2017. Complete genome sequence of a Shiga toxin-producing Enterobacter cloacae clinical isolate. Genome Announcements 5:e00883-17. doi:10.1128/genomeA.00883-17. 28912313 PMC5597754

