# Supplementary material for: A Global Survey of Hypervirulent Aeromonas hydrophila (vAh) Identified vAh Strains in the Lower Mekong River Basin and Diverse Opportunistic Pathogens from Farmed Fish and Other Environmental Sources
Source: Microbiol Spectr. 2023 Feb 23;11(2):e03705-22. doi: 10.1128/spectrum.03705-22 (PMC10101000; doi:10.1128/spectrum.03705-22)
Supplement: Supplemental file 1 — Fig. S1. Download spectrum.03705-22-s0001.pdf, PDF file, 2.2 MB [file spectrum.03705-22-s0001.pdf]

Supplemental Figure Legends

Figure S1A. Maximum likelihood phylogeny of vAh, non-vAh, *myo*-inositol utilizing *Aeromonas* spp., *E. cloacae* isolates, based on the IolA among the isolates. The *myo*-inositol utilizing *Aeromonas* spp. and vAh isolates that had significant alignment with vAh ML09-119 were inferred using maximum likelihood method for the evolutionary relationships, based on the amino acid sequences of IolA. The phylogeny test was a bootstrap method with 1000 bootstrap replications, the bootstrap of each clade was shown in a circle. The strains isolated from the same country were labeled in the same color. The protein is IolA (CoA-acylating methylmalonate-semialdehyde dehydrogenase), oxidizing malonate semialdehyde and methylmalonate semialdehyde to produce acetyl-CoA and propionyl-CoA.

Figure S1B. Maximum likelihood phylogeny of vAh, non-vAh, *myo*-inositol utilizing *Aeromonas* spp., *E. cloacae* isolates, based on the IolC among the isolates. The *myo*-inositol utilizing *Aeromonas* spp. and vAh isolates that had significant alignment with vAh ML09-119 were inferred using maximum likelihood method for the evolutionary relationships, based on the amino acid sequences of IolC. The phylogeny test was a bootstrap method with 1000 bootstrap replications, the bootstrap of each clade was shown in a circle. The strains isolated from the same country were labeled in the same color. The protein is IolC (5-dehydro-2-deoxygluconokinase), responsible for the generation of 6-phospho-5-dehydro-2-deoxy-D-gluconate from 5-dehydro-2-deoxy-D-gluconate by phosphorylation.

Figure S1C. Maximum likelihood phylogeny of vAh, non-vAh, *myo*-inositol utilizing *Aeromonas* spp., *E. cloacae* isolates, based on the IolD among the isolates. The *myo*-inositol utilizing *Aeromonas* spp. and vAh isolates that had significant alignment with vAh ML09-119 were inferred using maximum likelihood method for the evolutionary relationships, based on the amino acid sequences of IolD. The phylogeny test was a bootstrap method with 1000 bootstrap replications, the bootstrap of each clade was shown in a circle. The strains isolated from the same country were labeled in the same color. The protein is IolD (3D-(3,5/4)-

trihydroxycyclohexane-1,2-dione acyl hydrolase), responsible for the generation of 5-deoxy-glucuronate from 3D-(3,5/4)-trihydroxycyclohexane-1,2-dione.

Figure S1D. Maximum likelihood phylogeny of vAh, non-vAh, *myo*-inositol utilizing *Aeromonas* spp., *E. cloacae* isolates, based on the IolE among the isolates. The *myo*-inositol utilizing *Aeromonas* spp. and vAh isolates that had significant alignment with vAh ML09-119 were inferred using maximum likelihood method for the evolutionary relationships, based on the amino acid sequences of IolE. The phylogeny test was a bootstrap method with 1000 bootstrap replications, the bootstrap of each clade was shown in a circle. The strains isolated from the same country were labeled in the same color. The protein is IolE (inosose dehydratase), responsible for the dehydration of *myo*-inositol.

Figure S1E. Maximum likelihood phylogeny of vAh, non-vAh, *myo*-inositol utilizing *Aeromonas* spp., *E. cloacae* isolates, based on the IolG among the isolates. The *myo*-inositol utilizing *Aeromonas* spp. and vAh isolates that had significant alignment with vAh ML09-119 were inferred using maximum likelihood method for the evolutionary relationships, based on the amino acid sequences of IolG. The phylogeny test was a bootstrap method with 1000 bootstrap replications, the bootstrap of each clade was shown in a circle. The strains isolated from the same country were labeled in the same color. The protein is IolG (inositol 2-dehydrogenase), responsible for the oxidation of *myo*-inositol, producing 2-keto-*myo*-inositol.

Figure S1F. Maximum likelihood phylogeny of vAh, non-vAh, *myo*-inositol utilizing *Aeromonas* spp., *E. cloacae* isolates, based on the InoE among the isolates. The *myo*-inositol utilizing *Aeromonas* spp. and vAh isolates that had significant alignment with vAh ML09-119 were inferred using maximum likelihood method for the evolutionary relationships, based on the amino acid sequences of InoE. The phylogeny test was a bootstrap method with 1000 bootstrap replications, the bootstrap of each clade was shown in a circle. The strains isolated from the same country were labeled in the same color. The protein is InoE (*myo*-

inositol ABC transporter).

Figure S1G. Maximum likelihood phylogeny of vAh, non-vAh, *myo*-inositol utilizing *Aeromonas* spp., *E. cloacae* isolates, based on the InoF among the isolates. The *myo*-inositol utilizing *Aeromonas* spp. and vAh isolates that had significant alignment with vAh ML09-119 were inferred using maximum likelihood method for the evolutionary relationships, based on the amino acid sequences of InoF. The phylogeny test was a bootstrap method with 1000 bootstrap replications, the bootstrap of each clade was shown in a circle. The strains isolated from the same country were labeled in the same color. The protein is InoF (*myo*-inositol ABC transporter), responsible for transmembrane transport.

Figure S1H. Maximum likelihood phylogeny of vAh, non-vAh, *myo*-inositol utilizing *Aeromonas* spp., *E. cloacae* isolates, based on the InoL among the isolates. The *myo*-inositol utilizing *Aeromonas* spp. and vAh isolates that had significant alignment with vAh ML09-119 were inferred using maximum likelihood method for the evolutionary relationships, based on the amino acid sequences of InoL. The phylogeny test was a bootstrap method with 1000 bootstrap replications, the bootstrap of each clade was shown in a circle. The strains isolated from the same country were labeled in the same color. The protein is InoL (*myo*-inositol ABC transporter), responsible for the activity of ATPase-coupled transmembrane transporter.

Figure S1A

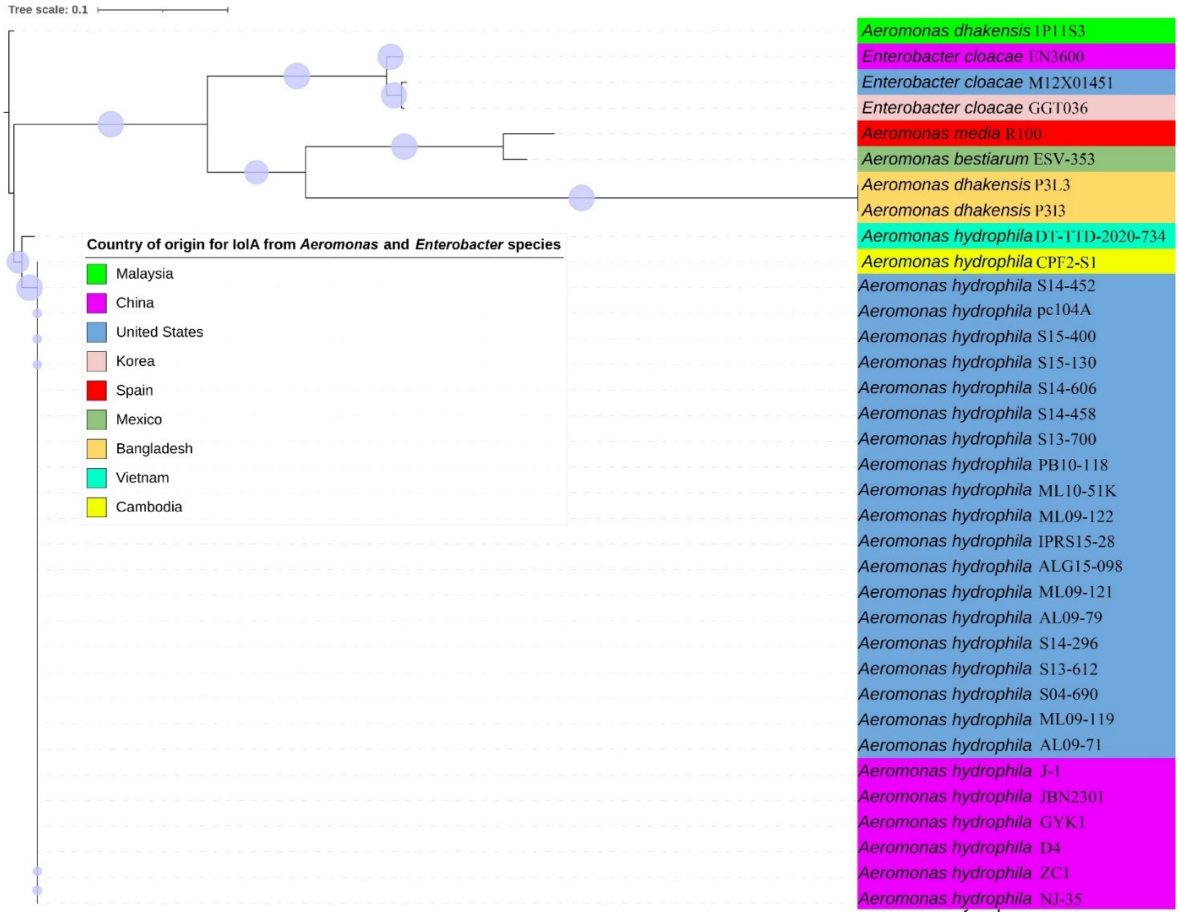

Tree scale: 0.1
Country of origin for lolA from Aeromonas and Enterobacter species
Malaysia
China
United States
Korea
Spain
Mexico
Bangladesh
Vietnam
Cambodia
Aeromonas dhakensis 1P11S3
Enterobacter cloacae EN3600
Enterobacter cloacae M12X01451
Enterobacter cloacae GGT036
Aeromonas media R100
Aeromonas bestiarum ESV-353
Aeromonas dhakensis P31.3
Aeromonas dhakensis P313
Aeromonas hydrophila DT-TTD-2020-734
Aeromonas hydrophila CPF2-S1
Aeromonas hydrophila S14-452
Aeromonas hydrophila pc104A
Aeromonas hydrophila S15-400
Aeromonas hydrophila S15-130
Aeromonas hydrophila S14-606
Aeromonas hydrophila S14-458
Aeromonas hydrophila S13-700
Aeromonas hydrophila PB10-118
Aeromonas hydrophila ML10-51K
Aeromonas hydrophila ML09-122
Aeromonas hydrophila IPRS15-28
Aeromonas hydrophila ALG15-098
Aeromonas hydrophila ML09-121
Aeromonas hydrophila AL09-79
Aeromonas hydrophila S14-296
Aeromonas hydrophila S13-612
Aeromonas hydrophila S04-690
Aeromonas hydrophila ML09-119
Aeromonas hydrophila AL09-71
Aeromonas hydrophila J-1
Aeromonas hydrophila JBN2301
Aeromonas hydrophila GYK1
Aeromonas hydrophila D4
Aeromonas hydrophila ZC1
Aeromonas hydrophila NJ-35

Figure S1B

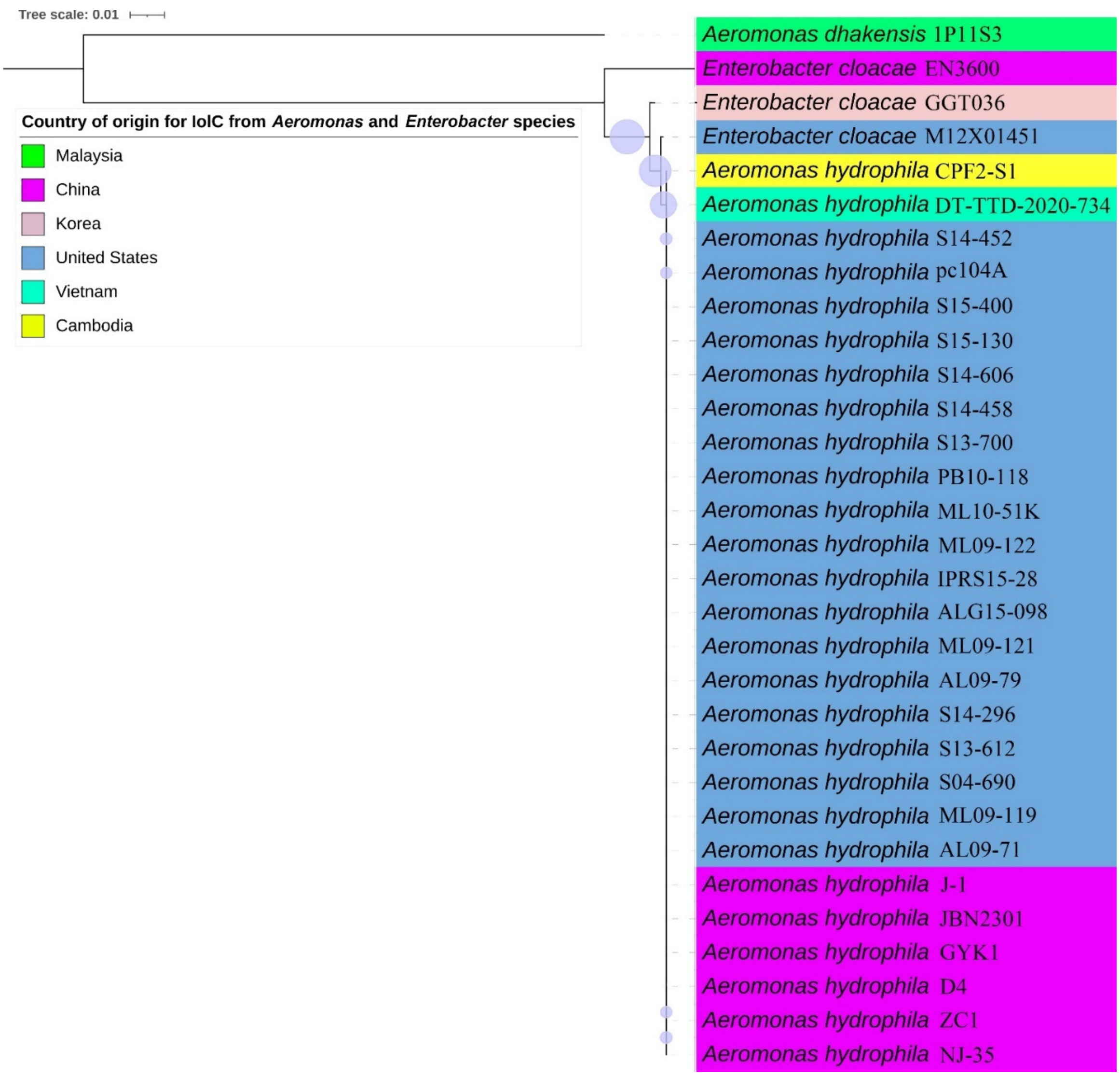
Tree scale: 0.01
Country of origin for IolC from Aeromonas and Enterobacter species
Malaysia
China
Korea
United States
Vietnam
Cambodia
Aeromonas dhakensis 1P11S3
Enterobacter cloacae EN3600
Enterobacter cloacae GGT036
Enterobacter cloacae M12X01451
Aeromonas hydrophila CPF2-S1
Aeromonas hydrophila DT-TTD-2020-734
Aeromonas hydrophila S14-452
Aeromonas hydrophila pc104A
Aeromonas hydrophila S15-400
Aeromonas hydrophila S15-130
Aeromonas hydrophila S14-606
Aeromonas hydrophila S14-458
Aeromonas hydrophila S13-700
Aeromonas hydrophila PB10-118
Aeromonas hydrophila ML10-51K
Aeromonas hydrophila ML09-122
Aeromonas hydrophila IPRS15-28
Aeromonas hydrophila ALG15-098
Aeromonas hydrophila ML09-121
Aeromonas hydrophila AL09-79
Aeromonas hydrophila S14-296
Aeromonas hydrophila S13-612
Aeromonas hydrophila S04-690
Aeromonas hydrophila ML09-119
Aeromonas hydrophila AL09-71
Aeromonas hydrophila J-1
Aeromonas hydrophila JBN2301
Aeromonas hydrophila GYK1
Aeromonas hydrophila D4
Aeromonas hydrophila ZC1
Aeromonas hydrophila NJ-35

Figure S1C

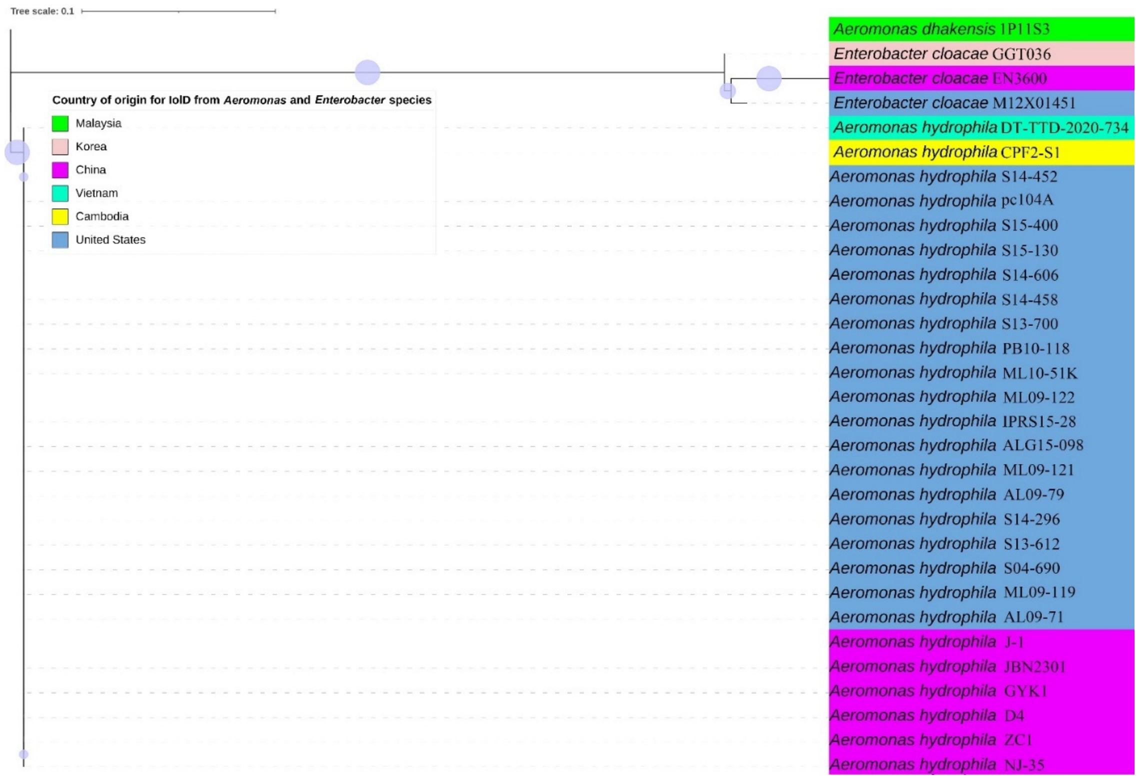

Tree scale: 0.1
Country of origin for IolD from *Aeromonas* and *Enterobacter* species
Malaysia
Korea
China
Vietnam
Cambodia
United States
Aeromonas dhakensis 1P11S3
Enterobacter cloacae GGT036
Enterobacter cloacae EN3600
Enterobacter cloacae M12X01451
Aeromonas hydrophila DT-TTD-2020-734
Aeromonas hydrophila CPF2-S1
Aeromonas hydrophila S14-452
Aeromonas hydrophila pc104A
Aeromonas hydrophila S15-400
Aeromonas hydrophila S15-130
Aeromonas hydrophila S14-606
Aeromonas hydrophila S14-458
Aeromonas hydrophila S13-700
Aeromonas hydrophila PB10-118
Aeromonas hydrophila ML10-51K
Aeromonas hydrophila ML09-122
Aeromonas hydrophila IPRS15-28
Aeromonas hydrophila ALG15-098
Aeromonas hydrophila ML09-121
Aeromonas hydrophila AL09-79
Aeromonas hydrophila S14-296
Aeromonas hydrophila S13-612
Aeromonas hydrophila S04-690
Aeromonas hydrophila ML09-119
Aeromonas hydrophila AL09-71
Aeromonas hydrophila J-1
Aeromonas hydrophila JBN2301
Aeromonas hydrophila GYK1
Aeromonas hydrophila D4
Aeromonas hydrophila ZC1
Aeromonas hydrophila NJ-35

Figure S1D

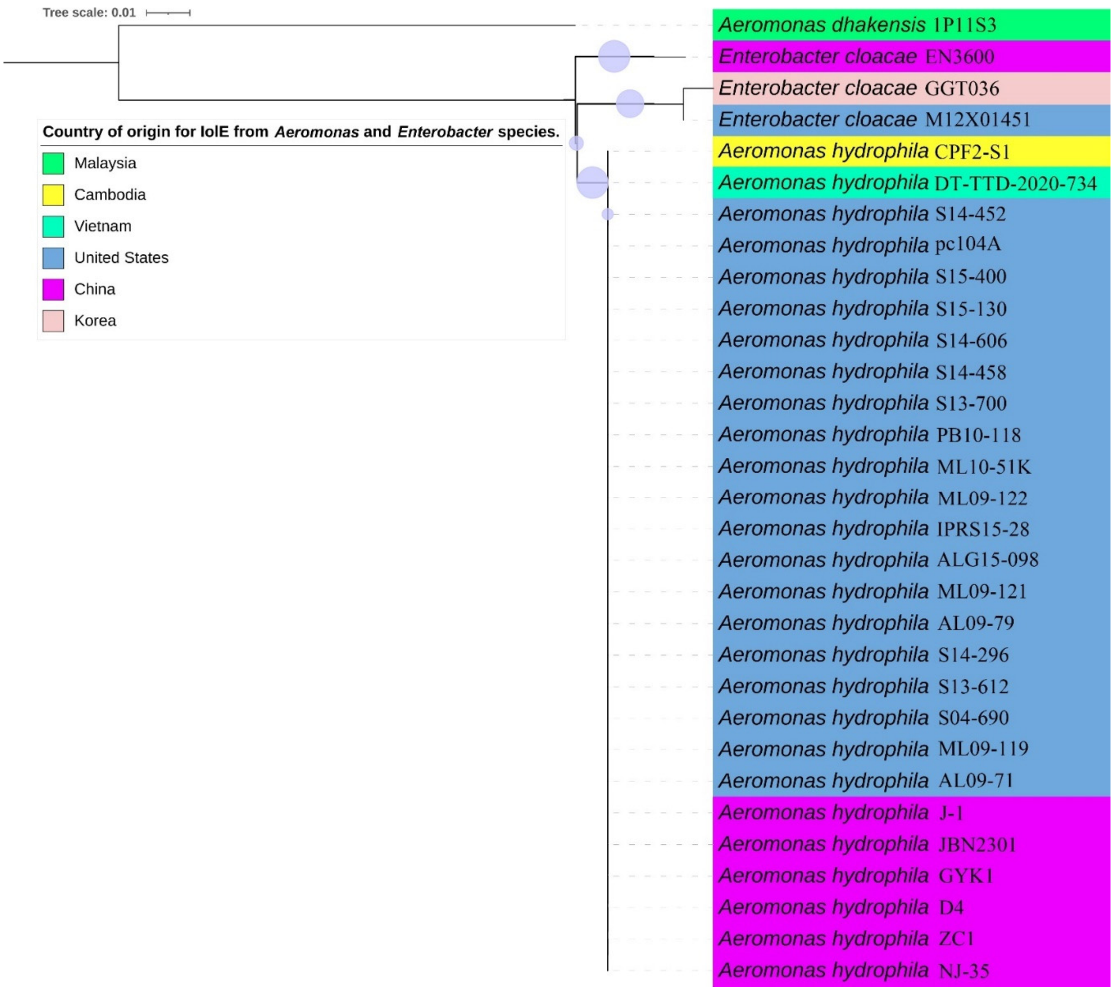

Tree scale: 0.01
Country of origin for IolE from Aeromonas and Enterobacter species.
Malaysia
Cambodia
Vietnam
United States
China
Korea
Aeromonas dhakensis 1P11S3
Enterobacter cloacae EN3600
Enterobacter cloacae GGT036
Enterobacter cloacae M12X01451
Aeromonas hydrophila CPF2-S1
Aeromonas hydrophila DT-TTD-2020-734
Aeromonas hydrophila S14-452
Aeromonas hydrophila pc104A
Aeromonas hydrophila S15-400
Aeromonas hydrophila S15-130
Aeromonas hydrophila S14-606
Aeromonas hydrophila S14-458
Aeromonas hydrophila S13-700
Aeromonas hydrophila PB10-118
Aeromonas hydrophila ML10-51K
Aeromonas hydrophila ML09-122
Aeromonas hydrophila IPRS15-28
Aeromonas hydrophila ALG15-098
Aeromonas hydrophila ML09-121
Aeromonas hydrophila AL09-79
Aeromonas hydrophila S14-296
Aeromonas hydrophila S13-612
Aeromonas hydrophila S04-690
Aeromonas hydrophila ML09-119
Aeromonas hydrophila AL09-71
Aeromonas hydrophila J-1
Aeromonas hydrophila JBN2301
Aeromonas hydrophila GYK1
Aeromonas hydrophila D4
Aeromonas hydrophila ZC1
Aeromonas hydrophila NJ-35

Figure S1E

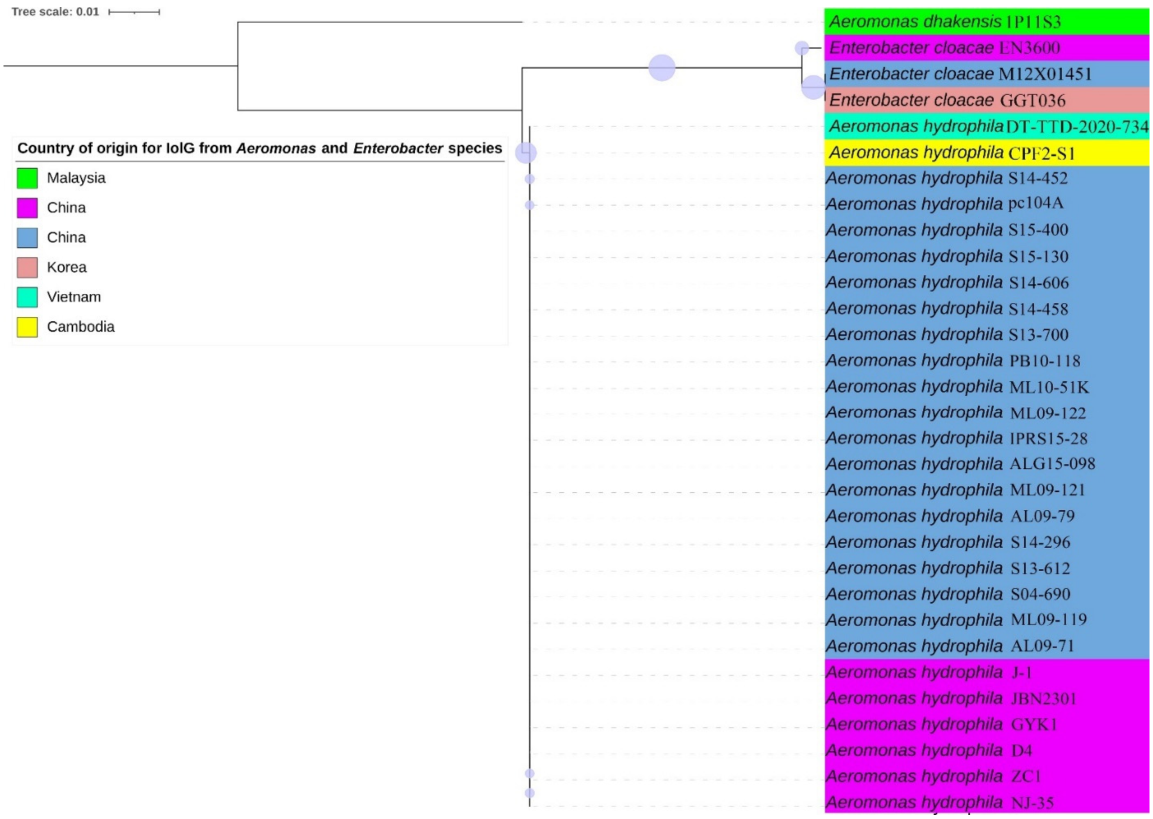

Tree scale: 0.01
Country of origin for IolG from *Aeromonas* and *Enterobacter* species
Malaysia
China
China
Korea
Vietnam
Cambodia
Aeromonas dhakensis 1P11S3
Enterobacter cloacae EN3600
Enterobacter cloacae M12X01451
Enterobacter cloacae GGT036
Aeromonas hydrophila DT-TTD-2020-734
Aeromonas hydrophila CPF2-S1
Aeromonas hydrophila S14-452
Aeromonas hydrophila pc104A
Aeromonas hydrophila S15-400
Aeromonas hydrophila S15-130
Aeromonas hydrophila S14-606
Aeromonas hydrophila S14-458
Aeromonas hydrophila S13-700
Aeromonas hydrophila PB10-118
Aeromonas hydrophila ML10-51K
Aeromonas hydrophila ML09-122
Aeromonas hydrophila IPRS15-28
Aeromonas hydrophila ALG15-098
Aeromonas hydrophila ML09-121
Aeromonas hydrophila AL09-79
Aeromonas hydrophila S14-296
Aeromonas hydrophila S13-612
Aeromonas hydrophila S04-690
Aeromonas hydrophila ML09-119
Aeromonas hydrophila AL09-71
Aeromonas hydrophila J-1
Aeromonas hydrophila JBN2301
Aeromonas hydrophila GYK1
Aeromonas hydrophila D4
Aeromonas hydrophila ZC1
Aeromonas hydrophila NJ-35

Figure S1F

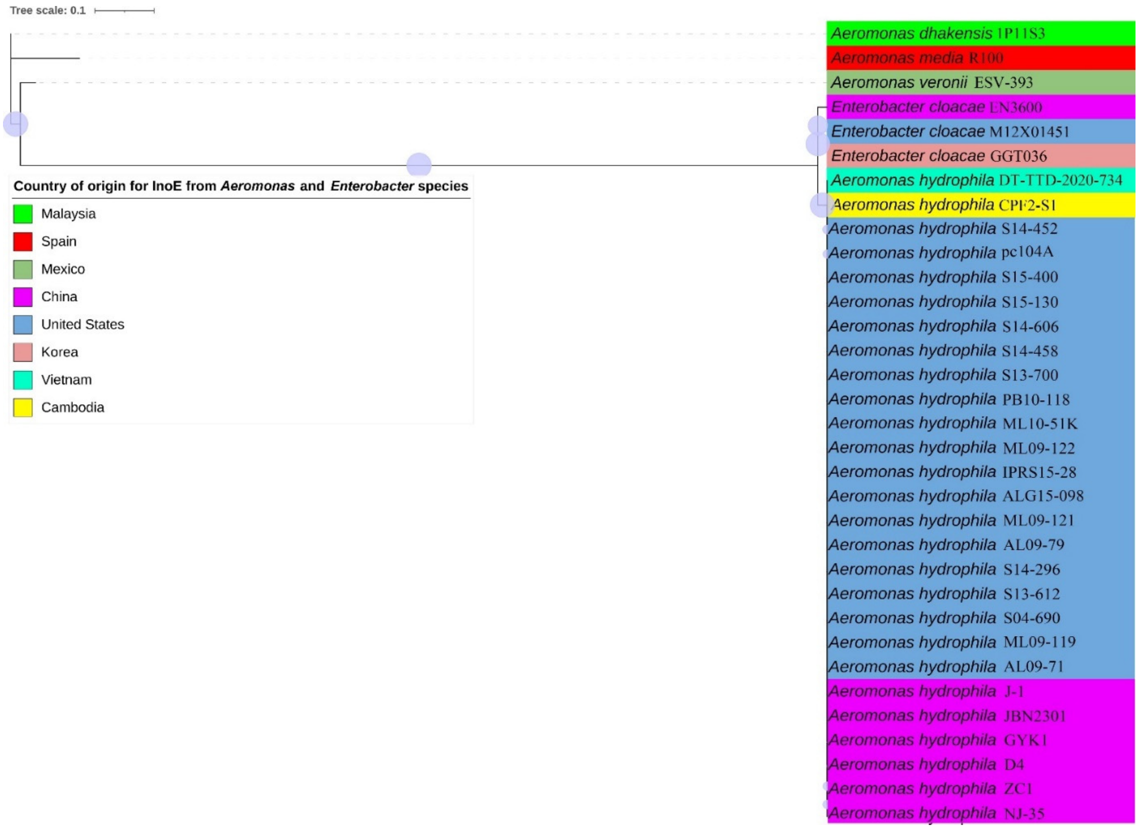
Tree scale: 0.1
Aeromonas dhakensis 1P11S3
Aeromonas media R100
Aeromonas veronii ESV-393
Enterobacter cloacae LN3600
Enterobacter cloacae M12X01451
Enterobacter cloacae GGT036
Aeromonas hydrophila DT-TTD-2020-734
Aeromonas hydrophila CPF2-S1
Aeromonas hydrophila S14-452
Aeromonas hydrophila pc104A
Aeromonas hydrophila S15-400
Aeromonas hydrophila S15-130
Aeromonas hydrophila S14-606
Aeromonas hydrophila S14-458
Aeromonas hydrophila S13-700
Aeromonas hydrophila PB10-118
Aeromonas hydrophila ML10-51K
Aeromonas hydrophila ML09-122
Aeromonas hydrophila IPRS15-28
Aeromonas hydrophila ALG15-098
Aeromonas hydrophila ML09-121
Aeromonas hydrophila AL09-79
Aeromonas hydrophila S14-296
Aeromonas hydrophila S13-612
Aeromonas hydrophila S04-690
Aeromonas hydrophila ML09-119
Aeromonas hydrophila AL09-71
Aeromonas hydrophila J-1
Aeromonas hydrophila JBN2301
Aeromonas hydrophila GYK1
Aeromonas hydrophila D4
Aeromonas hydrophila ZC1
Aeromonas hydrophila NJ-35
Country of origin for InoE from Aeromonas and Enterobacter species
Malaysia
Spain
Mexico
China
United States
Korea
Vietnam
Cambodia

Figure S1G

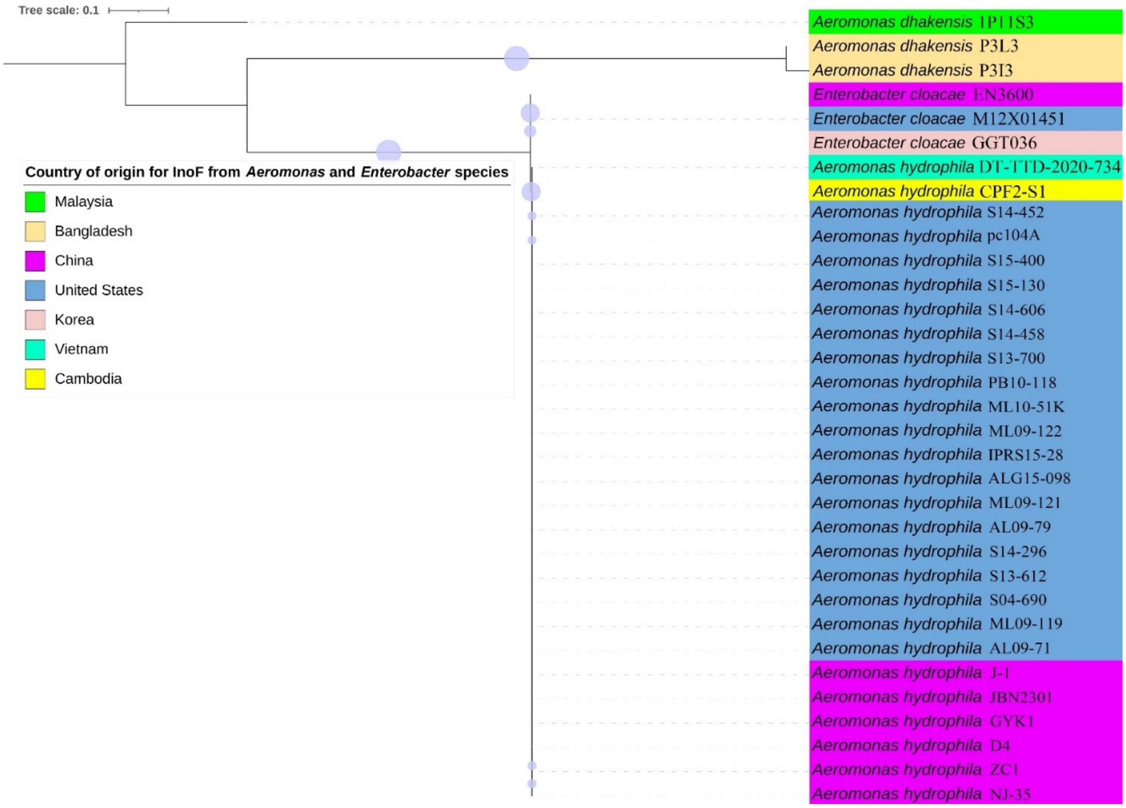

Tree scale: 0.1
Country of origin for InoF from Aeromonas and Enterobacter species
Malaysia
Bangladesh
China
United States
Korea
Vietnam
Cambodia
Aeromonas dhakensis 1P11S3
Aeromonas dhakensis P3L3
Aeromonas dhakensis P3I3
Enterobacter cloacae EN3600
Enterobacter cloacae M12X01451
Enterobacter cloacae GGT036
Aeromonas hydrophila DT-TTD-2020-734
Aeromonas hydrophila CPF2-S1
Aeromonas hydrophila S14-452
Aeromonas hydrophila pc104A
Aeromonas hydrophila S15-400
Aeromonas hydrophila S15-130
Aeromonas hydrophila S14-606
Aeromonas hydrophila S14-458
Aeromonas hydrophila S13-700
Aeromonas hydrophila PB10-118
Aeromonas hydrophila ML10-51K
Aeromonas hydrophila ML09-122
Aeromonas hydrophila IPRS15-28
Aeromonas hydrophila ALG15-098
Aeromonas hydrophila ML09-121
Aeromonas hydrophila AL09-79
Aeromonas hydrophila S14-296
Aeromonas hydrophila S13-612
Aeromonas hydrophila S04-690
Aeromonas hydrophila ML09-119
Aeromonas hydrophila AL09-71
Aeromonas hydrophila J-1
Aeromonas hydrophila JBN2301
Aeromonas hydrophila GYK1
Aeromonas hydrophila D4
Aeromonas hydrophila ZC1
Aeromonas hydrophila NJ-35

Figure S1H

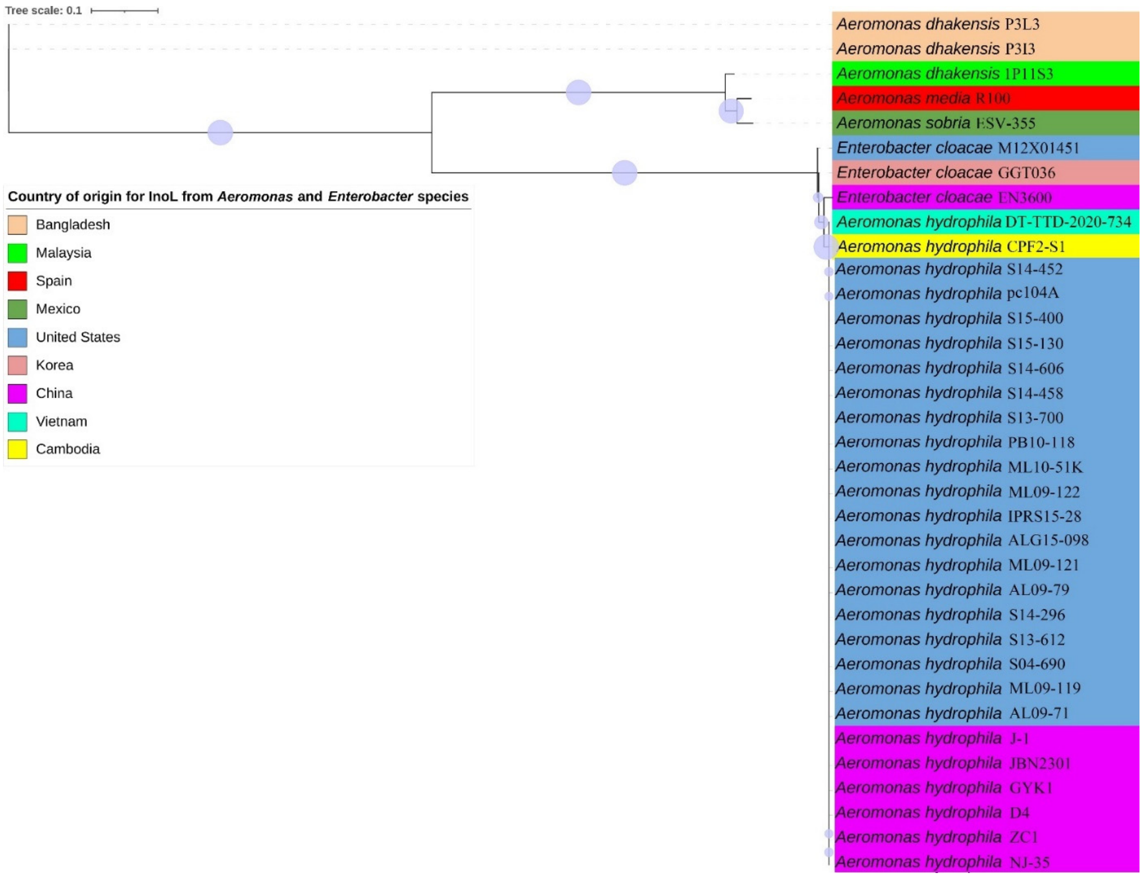

Tree scale: 0.1
Aeromonas dhakensis P3L3
Aeromonas dhakensis P3I3
Aeromonas dhakensis 1P11S3
Aeromonas media R100
Aeromonas sobria ESV-355
Enterobacter cloacae M12X01451
Enterobacter cloacae GGT036
Enterobacter cloacae EN3600
Aeromonas hydrophila DT-TTD-2020-734
Aeromonas hydrophila CPF2-S1
Aeromonas hydrophila S14-452
Aeromonas hydrophila pc104A
Aeromonas hydrophila S15-400
Aeromonas hydrophila S15-130
Aeromonas hydrophila S14-606
Aeromonas hydrophila S14-458
Aeromonas hydrophila S13-700
Aeromonas hydrophila PB10-118
Aeromonas hydrophila ML10-51K
Aeromonas hydrophila ML09-122
Aeromonas hydrophila IPRS15-28
Aeromonas hydrophila ALG15-098
Aeromonas hydrophila ML09-121
Aeromonas hydrophila AL09-79
Aeromonas hydrophila S14-296
Aeromonas hydrophila S13-612
Aeromonas hydrophila S04-690
Aeromonas hydrophila ML09-119
Aeromonas hydrophila AL09-71
Aeromonas hydrophila J-1
Aeromonas hydrophila JBN2301
Aeromonas hydrophila GYK1
Aeromonas hydrophila D4
Aeromonas hydrophila ZC1
Aeromonas hydrophila NJ-35
Country of origin for InoL from Aeromonas and Enterobacter species
Bangladesh
Malaysia
Spain
Mexico
United States
Korea
China
Vietnam
Cambodia
